# MicroRNA-146 represses endothelial activation by inhibiting pro-inflammatory pathways

**DOI:** 10.1002/emmm.201202318

**Published:** 2013-06-03

**Authors:** Henry S Cheng, Nirojini Sivachandran, Andrew Lau, Emilie Boudreau, Jimmy L Zhao, David Baltimore, Paul Delgado-Olguin, Myron I Cybulsky, Jason E Fish

**Affiliations:** 1 Toronto General Research Institute, University Health NetworkToronto, Canada; 2 Department of Laboratory Medicine and Pathobiology, University of TorontoToronto, Canada; 3 The Heart and Stroke Richard Lewar Centre of Excellence in Cardiovascular ResearchToronto, Canada; 4 Division of Biology, California Institute of TechnologyPasadena, CA; 5 Department of Physiology & Experimental Medicine, The Hospital for Sick ChildrenToronto, Canada; 6 Department of Molecular Genetics, University of TorontoToronto, Canada

**Keywords:** atherosclerosis, endothelium, gene regulation, inflammation, microRNA

## Abstract

Activation of inflammatory pathways in the endothelium contributes to vascular diseases, including sepsis and atherosclerosis. We demonstrate that miR-146a and miR-146b are induced in endothelial cells upon exposure to pro-inflammatory cytokines. Despite the rapid transcriptional induction of the *miR-146a/b* loci, which is in part mediated by EGR-3, miR-146a/b induction is delayed and sustained compared to the expression of leukocyte adhesion molecules, and in fact coincides with the down-regulation of inflammatory gene expression. We demonstrate that miR-146 negatively regulates inflammation. Over-expression of miR-146a blunts endothelial activation, while knock-down of miR-146a/b *in vitro* or deletion of *miR-146a* in mice has the opposite effect. MiR-146 represses the pro-inflammatory NF-κB pathway as well as the MAP kinase pathway and downstream EGR transcription factors. Finally, we demonstrate that HuR, an RNA binding protein that promotes endothelial activation by suppressing expression of endothelial nitric oxide synthase (eNOS), is a novel miR-146 target. Thus, we uncover an important negative feedback regulatory loop that controls pro-inflammatory signalling in endothelial cells that may impact vascular inflammatory diseases.

## INTRODUCTION

The endothelium plays a central role in the pathogenesis of vascular inflammatory diseases such as sepsis (Aird, 2003) and atherosclerosis (Gimbrone & Garcia-Cardena, 2012; Pober & Sessa, 2007). During sepsis, massive circulating levels of pro-inflammatory cytokines activate the endothelium and drive pathological vascular permeability and tissue oedema, which lead to acute organ dysfunction (Aird, 2003). Blocking endothelial activation can limit mortality in mouse models of sepsis (London et al, 2010). Endothelial activation also plays a pervasive role in atherosclerosis, a chronic vascular inflammatory disorder that is characterized by vessel narrowing, thrombosis and occlusion (Gimbrone & Garcia-Cardena, 2012; Pober & Sessa, 2007). Cell-surface expression of leukocyte adhesion molecules such as vascular cell adhesion molecule-1 (VCAM-1), intercellular adhesion molecule-1 (ICAM-1) and E-Selectin, and secretion of chemokines such as monocyte chemoattractant protein-1 (MCP-1), facilitates the binding of circulating monocytes to the blood vessel wall. Following transmigration into the intima, these cells mature into inflammatory macrophages, and their secretion of pro-inflammatory cytokines further promotes endothelial activation, and serves to drive a feed-forward loop that perpetuates leukocyte recruitment (Pober & Sessa, 2007). Identifying molecules that negatively regulate inflammatory pathways in the endothelium may provide novel therapeutic targets for the treatment of acute or chronic vascular inflammatory diseases.

Activation of pro-inflammatory transcriptional programs such as the NF-κB signalling pathway (Gareus et al, 2008; Ruland, 2011; Ye et al, 2008) and the mitogen-activated protein kinase (MAPK)/early growth response (EGR) pathway (Hajra et al, 2000; Shin et al, 2009; Wieland et al, 2005; Yan et al, 2000) can cooperatively drive endothelial activation and vascular inflammation. Considering that prolonged or exaggerated inflammatory responses are detrimental, it is not surprising that cellular negative feedback loops act to control the duration and intensity of an inflammatory response (Ruland, 2011). For example, activation of the NF-κB pathway leads to the induction of IκB proteins, which bind to NF-κB proteins in the nucleus and exports them to the cytoplasm (Arenzana-Seisdedos et al, 1995). EGR transcription factors also induce the expression of their own repressor proteins (Kumbrink et al, 2005). In addition to feedback regulation at the level of transcription, microRNAs have recently been identified that serve in post-transcriptional negative feedback loops that modulate inflammatory signalling. MicroRNAs bind to the 3′ UTRs of target mRNAs and inhibit their translation and/or stability (Bartel, 2009). *MiR-146a* was previously found to be transcriptionally induced by NF-κB in response to activation of innate immune signalling in monocytes (Taganov et al, 2006). MiR-146a targets the adaptor proteins TRAF6 and IRAK1/2 (Bhaumik et al, 2008; Hou et al, 2009; Nahid et al, 2009; Taganov et al, 2006) and can inhibit activation of the NF-κB pathway (Bhaumik et al, 2008; Zhao et al, 2011), suggesting that miR-146a participates in a negative feedback loop to control NF-κB signalling in monocytes. However, the function of miR-146a/b is poorly understood in endothelial cells.

We previously identified miR-146a and miR-146b as being enriched in endothelial cells isolated from differentiating embryonic stem cells (Fish et al, 2008). Herein we demonstrate that *miR-146a* and *miR-146b* are enriched in endothelial cells *in vivo* and that they are strongly induced in endothelial cells in response to pro-inflammatory cytokines. We also identify a novel transcriptional pathway involving EGR proteins that participates in the induction of *miR-146a* and *miR-146b*. Through delayed induction kinetics, miR-146a/b appear to act as negative feedback regulators of inflammatory signalling in endothelial cells. MiR-146 inhibits endothelial activation by dampening the activation of pro-inflammatory transcriptional programs, including the NF-κB, AP-1 and MAPK/EGR pathways, likely through regulation of IL-1β signalling pathway adaptor proteins (*i.e*., TRAF6, IRAK1/2). In addition, miR-146 modulates post-transcriptional pro-inflammatory pathways via targeting of the RNA binding protein HuR. We provide evidence that HuR facilitates endothelial activation by repressing expression of endothelial nitric oxide synthase (eNOS), a major source of nitric oxide, which potently inhibits leukocyte adhesion (Kubes et al, 1991). Thus miR-146 represses both transcriptional and post-transcriptional activation of the inflammatory program. Our results reveal a potent anti-inflammatory action of miR-146a/b in the endothelium and suggest that therapeutic elevation of this microRNA family may be a useful treatment strategy for vascular inflammatory diseases, including sepsis and atherosclerosis.

## RESULTS

### Induction of miR-146a and miR-146b (miR-146a/b) by inflammatory stimuli in endothelial cells

**Figure 1:**
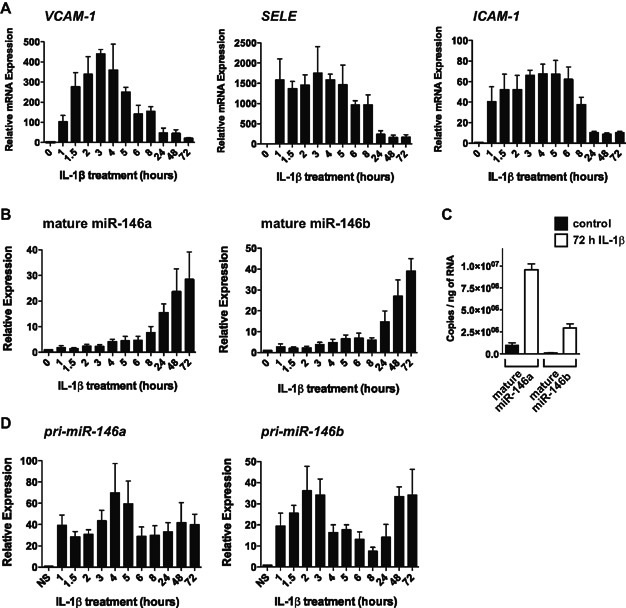
MiR-146a and miR-146b are induced in response to interleukin-1β (IL-1β) treatment of endothelial cells Levels of pro-inflammatory genes (*VCAM-1*, *SELE* (E-Selectin), *ICAM1*) were measured in IL-1β-treated human umbilical vein endothelial cells (HUVEC) by quantitative reverse transcriptase real-time PCR (qRT-PCR), revealing that these inflammatory genes were rapidly induced by IL-1β, but decreased by 24 h (h). Data represent the mean ± SEM of three independent experiments.Levels of mature miR-146a and miR-146b were assessed by qRT-PCR (*n* = 3). MiR-146a/b were increased following prolonged treatment with IL-1β.The copy numbers of miR-146a and miR-146b were quantified in non-stimulated (NS) and 72 h IL-1β-treated endothelial cells (*n* = 3).Assessment of the primary transcripts (pri-cursors), *pri-miR-146a* and *pri-miR-146b*, by qRT-PCR demonstrated rapid transcriptional up-regulation, which mirrored that of other inflammatory genes (*n* = 5). The transcription of *miR-146a/b* appeared to be sustained during prolonged inflammation.

Treatment of human umbilical vein endothelial cells (HUVEC) with the pro-inflammatory cytokine, IL-1β, induced the rapid induction of mRNAs encoding leukocyte adhesion molecules, such as VCAM-1, E-Selectin and ICAM1 (Fig 1A). We next measured levels of miR-146a and miR-146b. Since miR-146a and miR-146b differ by only two nucleotides near their 3′ ends, we designed primers that amplified only miR-146a or miR-146b (see Materials and Methods Section). We found that these microRNAs were dramatically induced in response to IL-1β treatment (Fig 1B). Similar induction of miR-146a/b was observed following tumor necrosis factor-α (TNF-α) treatment (Supporting Information Fig S1). MiR-146a/b levels were increased during the late stages of an inflammatory response (*i.e*., 8–72 h post-treatment), but levels were only modestly elevated at early stages (*i.e*., 1–4 h post-treatment; Fig 1, Supporting Information Fig S1). Interestingly, the induction of miR-146a/b coincided with the down-regulation of adhesion molecule genes (Fig 1A and B). Quantification of miR-146a/b levels revealed that miR-146a was expressed ∼9-fold higher than miR-146b in unstimulated cells, and ∼3-fold higher than miR-146b in IL-1β-treated cells (Fig 1C). We next measured the transcription of the *miR-146a* and *miR-146b* genomic loci. *MiR-146a* is processed from a two-exon non-protein coding mRNA transcript on chromosome 5, and we therefore measured unspliced pre-mRNA of this transcript as a surrogate of transcription [as we have done previously (Fish et al, 2010)]. *MiR-146b* is processed from a single exon transcript on chromosome 10, and primers were designed to measure the levels of this transcript. We found that transcription of *miR-146a* and *miR-146b* were rapidly (within 1 h) and dramatically (40- and 20-fold, respectively) induced in response to IL-1β (Fig 1D). The transcription of *miR-146a* and *miR-146b* was sustained for the duration of IL-1β treatment. This is in contrast to *VCAM-1*, *SELE* (E-Selectin) and *ICAM-1* mRNA, which were down-regulated after 8 h of IL-1β treatment. Despite the rapid transcription of the *miR-146a* and *miR-146b* genes, delayed expression of mature microRNAs implies inefficient or delayed processing of *miR-146a/b* in cytokine-stimulated endothelial cells.

### MiR-146a/b expression is sustained following removal of pro-inflammatory cytokines

**Figure 2:**
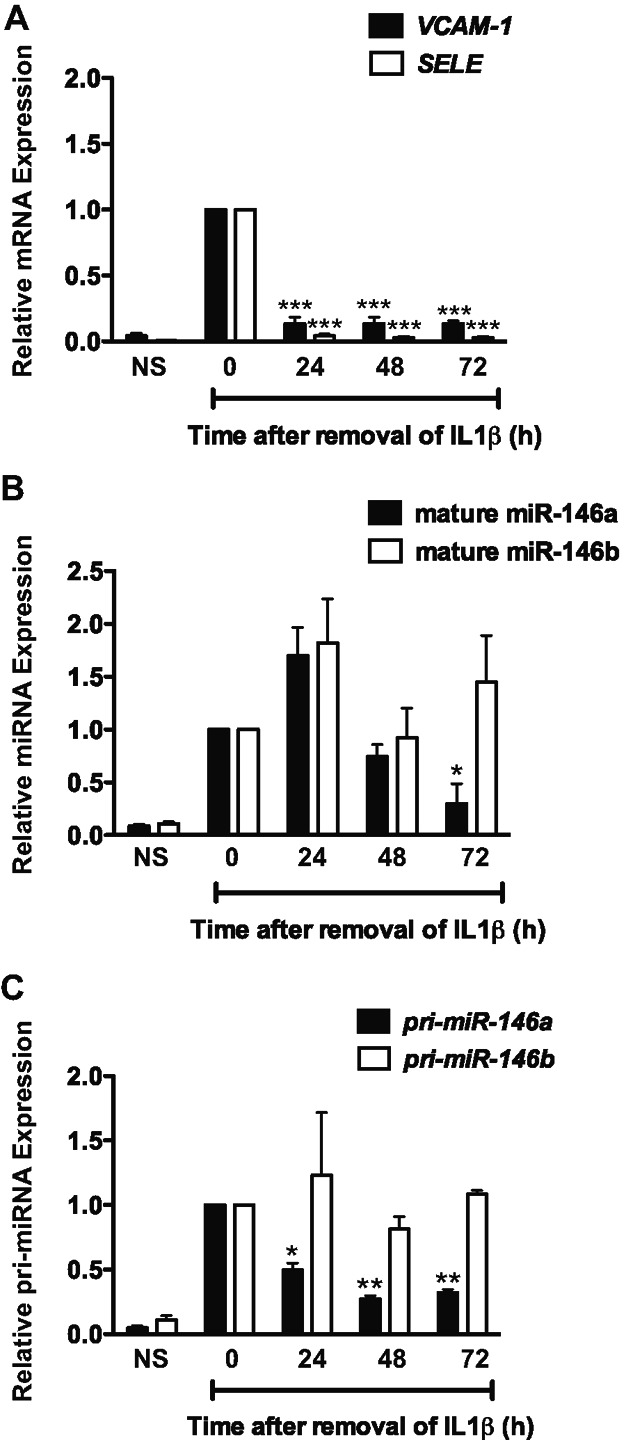
MiR-146a and miR-146b expression is sustained after the removal of IL-1β Endothelial cells were treated with IL-1β for 24 h, after which IL-1β was removed. Levels of *VCAM-1* and *SELE* (A), mature miR-146a/b (B) and *pri-miR-146a/b* (C) were monitored at various time-points after the removal of IL-1β by qRT-PCR. While *VCAM-1* and *SELE* rapidly returned to base-line levels, miR-146a/b levels remained elevated. The transcription of *miR-146a* decreased by 24 h after removal of IL-1β, while transcription of *miR-146b* was sustained in the absence of IL-1β. Data represents the mean ± SEM of three independent experiments. Statistical analyses were performed using *t*-test to compare post-IL-1β removal time-points to 24 h of IL-1β treatment (*i.e*., time zero). Significant *p*-values (from left to right) in (A) are 0.0004, <0.0001, 0.0004, <0.0001, 0.0006 and 0.0001, respectively. *p*-value in (B) is 0.049. *p*-Values in (C) are 0.012, 0.001 and 0.001, respectively.

To determine the stability of the IL-1β-mediated induction of miR-146a/b we treated endothelial cells with IL-1β for 24 h and then removed the cytokine. In contrast to inflammatory genes such as *VCAM-1* and *SELE*, which were rapidly down-regulated upon removal of IL-1β (Fig 2A), miR-146a/b remained elevated for more than 2 days (Fig 2B). MiR-146b expression was especially long-lived. While the levels of *pri-miR-146a* decreased following the removal of IL-1β, levels of *pri-miR-146b* remained unchanged, suggesting that the transcription of the *miR-146b* locus is maintained following the removal of pro-inflammatory cytokines (Fig 2C). The induction of miR-146a/b by IL-1β therefore appears to be highly stable, even in the absence of the initiating stimulus.

### Over-expression of miR-146a inhibits the endothelial inflammatory response

**Figure 3:**
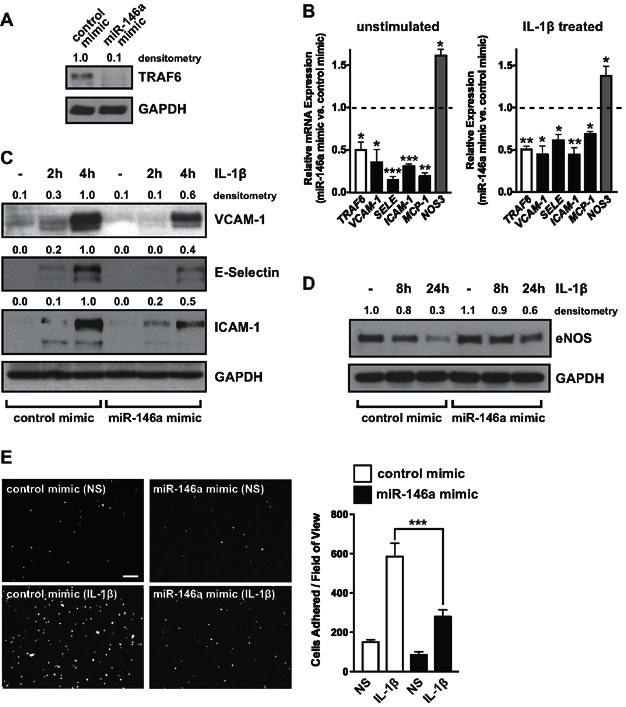
MiR-146a over-expression represses the endothelial inflammatory response MiR-146a was over-expressed in endothelial cells by transfection of miR-146a mimic and levels of a known target of miR-146, TRAF6, were assessed by Western blot. GAPDH was used as a loading control and densitometry is indicated above. A representative experiment is shown.Expression of *TRAF6* (white bar), inflammatory genes (*VCAM-1*, *ICAM1*, *SELE* (E-Selectin), and *MCP-1*; black bars), as well as *NOS3* (eNOS; grey bar), were measured in unstimulated (left) and IL-1β-stimulated cells (right) by qRT-PCR. For inflammatory genes, gene expression was analysed 1.5 h after addition of IL-1β, while *NOS3* was assessed after 8 h. Data is presented as mRNA levels in miR-146a mimic-transfected cells compared to control mimic-transfected cells, with the dotted line indicating a ratio of 1 (*i.e*., no change; *n* = 4). *p* Values (*t*-test) from left to right are 0.031, 0.023, 0.0002, 0.0001, 0.002, 0.014, 0.006, 0.012, 0.012, 0.006, 0.011 and 0.045, respectively.Western blotting was performed to measure expression of VCAM-1, E-Selectin and ICAM-1 protein in control and miR-146a mimic-transfected cells. Densitometry is indicated.Western blotting of eNOS protein was performed in control and miR-146a mimic-transfected cells.Adhesion of the mononuclear cell line, THP-1, to unstimulated and IL-1β-treated endothelial cells transfected with control or miR-146a mimic was visualized (left) and quantified (right), revealing a strong anti-adhesive effect of miR-146a over-expression. Scale bar is 200 μm. Shown is a representative experiment (mean ± SEM) with three replicate wells and three images per well for each condition. ANOVA, *p* < 0.0001. ***Indicates a significant difference between IL-1β-treated control and miR-146a mimic-transfected cells, *p* < 0.001.

To assess the function of elevated levels of miR-146 in endothelial cells, we over-expressed miR-146a via transfection of miR-146a mimic. Over-expression of miR-146a in HUVEC resulted in decreased expression of TRAF6 (Fig 3A), a known target of miR-146 (Taganov et al, 2006). Next we assessed the expression of several pro-inflammatory genes (*VCAM-1*, *ICAM-1*, *SELE* and *MCP-1*) by qRT-PCR, and found that the basal levels of these mRNAs were suppressed in unstimulated miR-146a over-expressing cells (Fig 3B, left). Importantly, miR-146a over-expression also dampened the induction of these inflammatory genes in response to IL-1β treatment (Fig 3B, right). Nitric oxide (NO) generated by eNOS potently inhibits leukocyte adhesion to the endothelium (Kubes et al, 1991), and eNOS (*NOS3*) is known to be transcriptionally (Anderson et al, 2004) and post-transcriptionally (Yoshizumi et al, 1993) repressed following treatment of endothelial cells with pro-inflammatory cytokines. The level of *NOS3* mRNA in unstimulated cells over-expressing miR-146a was elevated (Fig 3B, left). After 8 h of IL-1β treatment, *NOS3* mRNA was decreased by 45.0 ± 6.5% (*p* = 0.004, not shown). Over-expression of miR-146a blunted this IL-1β-dependent decrease in *NOS3* mRNA levels (Fig 3B, right). Western blotting confirmed that the induction of VCAM-1, E-Selectin and ICAM-1 protein was inhibited in miR-146a over-expressing cells (Fig 3C), and that the loss of eNOS expression was blunted (Fig 3D). Consistent with a reduction in inducible adhesion molecule expression and an increase in eNOS protein, miR-146a over-expression in HUVEC reduced the number of THP-1 monocytes that adhered to IL-1β-treated endothelial cells (Fig 3E). Over-expression of miR-146a in aortic endothelial cells also inhibited leukocyte adhesion (Supporting Information Fig S2), suggesting that miR-146a broadly represses endothelial activation. MiR-146a therefore inhibits the endothelial inflammatory response, including the induction of adhesion molecules and chemoattractants and the loss of eNOS expression.

### Endogenous miR-146 inhibits the endothelial inflammatory response

**Figure 4:**
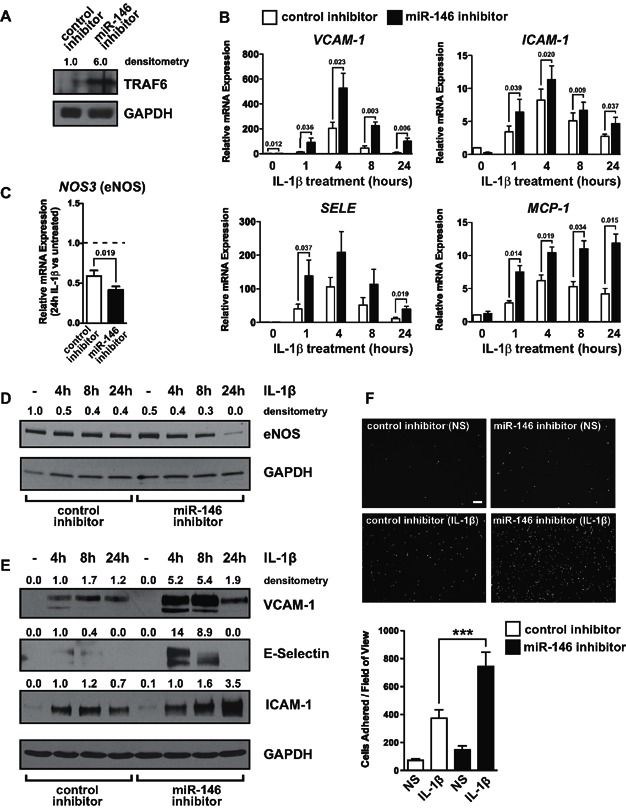
Endogenous miR-146 restrains endothelial activation Endothelial cells were transfected with a miR-146 LNA inhibitor (which reduces levels of miR-146a and miR-146b by >80%), and the level of a known target of miR-146, TRAF6, was measured by Western blot.The expression of inflammatory genes (*VCAM-1*, *ICAM-1*, *SELE*, and *MCP-1*) in unstimulated and IL-1β-stimulated cells was assessed by qRT-PCR. Data represents mean ± SEM of three independent experiments. Significant *p* values (*t*-test) are indicated above.Levels of *NOS3* mRNA were assessed by qRT-PCR in control inhibitor and miR-146 inhibitor transfected cells after 24 h of IL-1β treatment (*n* = 3). Data is expressed relative to untreated cells.Levels of eNOS protein were measured in control and miR-146 inhibitor transfected cells.Western blotting was performed to measure VCAM-1, E-Selectin and ICAM-1 protein expression in control inhibitor and miR-146 inhibitor transfected cells.Monocyte adhesion assays were performed in control and miR-146 inhibitor transfected endothelial cells. Representative images are shown (above) and quantification of a representative experiment (three replicate wells, three images per well) is shown (below). Scale bar is 200 μm. ANOVA, *p* < 0.0001. ***Indicates a significant difference between IL-1β-treated control and miR-146 inhibitor-transfected cells, *p* < 0.001.

We next utilized a miR-146 locked-nucleic acid (LNA) inhibitor to assess the function of endogenous miR-146 in endothelial cells. In addition to reducing the level of mature miR-146a by 81.7 ± 6.5%, this inhibitor also reduced the level of miR-146b by 92.5 ± 2.7% (not shown), likely owing to the limited (two nucleotide) difference in sequence between miR-146a and miR-146b. Treatment with miR-146 inhibitor elevated the level of the miR-146 target, TRAF6 (Fig 4A). Additionally, miR-146 inhibitor increased the basal levels of *VCAM-1* mRNA, and had a potent effect on the IL-1β-inducible expression of *VCAM-1*, *ICAM-1*, *SELE* and *MCP-1* (Fig 4B). Endogenous miR-146 appeared to restrain the intensity as well as the duration of the inflammatory response, since these inflammatory genes remained at elevated levels 24 h after IL-1β treatment (Fig 4B). In addition, the decrease in eNOS (*NOS3*) mRNA that was observed after a 24 h treatment with IL-1β was augmented by miR-146 inhibitor (Fig 4C). Western blotting confirmed that the loss of eNOS protein in response to IL-1β treatment was enhanced by miR-146 inhibition (Fig 4D) and that IL-1β-inducible VCAM-1, E-Selectin and ICAM-1 protein expression was greatly enhanced by miR-146 inhibition (Fig 4E). Finally, inhibition of miR-146 in endothelial cells enhanced the adhesion of THP-1 monocytes following IL-1β treatment (Fig 4F).

### MiR-146 negatively regulates the NF-κB, AP-1 and MAPK/early growth response (EGR) pathways

**Figure 5:**
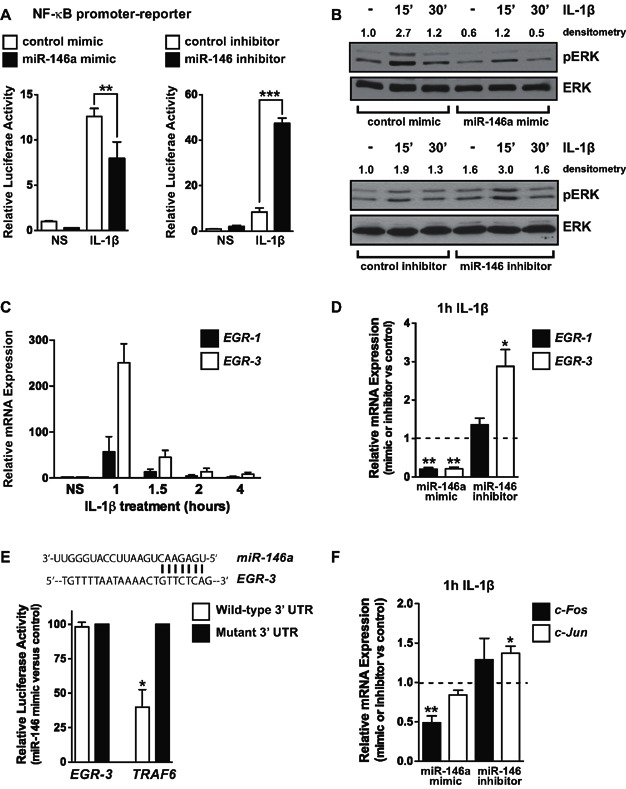
MiR-146 inhibits the induction of NF-κB, MAPK/EGR and AP-1 pathways The activity of a NF-κB promoter-luciferase reporter construct was assessed in endothelial cells transfected with control mimic, miR-146a mimic, control inhibitor or miR-146 inhibitor. MiR-146a over-expression reduced IL-1β-induced NF-κB-dependent promoter activity, while inhibition of miR-146 enhanced activity. Data represents the mean ± SEM of three independent experiments. ANOVA, *p* < 0.0001 for mimic and inhibitor data. ** and *** indicate a significant difference between the indicated groups, *p* < 0.01 and *p* < 0.001, respectively.Activation of the MAP kinase pathway was assessed by measuring the levels of phosphorylated ERK (pERK) (p42/p44). Total levels of ERK2 were used as a loading control. MiR-146a over-expression inhibited the basal and IL-1β-induced levels of pERK, while miR-146 inhibitor had the opposite effect.Induction of *EGR-1* and *EGR-3* in response to IL-1β was assessed by qRT-PCR, demonstrating rapid and transient induction (*n* = 3).MiR-146a over-expression inhibited the IL-1β-mediated induction of *EGR-1* and *EGR-3*, while inhibition of miR-146 enhanced the induction of *EGR-3* (*n* = 3). Significant *p* values (*t*-test) from left to right are 0.002, 0.004 and 0.022, respectively.Schematic of a potential miR-146 binding site in the 3′ UTR of *EGR-3* (top). Luciferase assays utilizing wild-type or seed-mutated *EGR-3* concatemer or *TRAF6* 3′ UTR sequences were performed in the presence of control or miR-146a mimic (*p* = 0.042, *t*-test, *n* = 3).Activation of the JNK/AP-1 pathway was assessed by measuring the induction of *c-Fos* and *c-Jun* by qRT-PCR. MiR-146a over-expression reduced c-Fos expression, while inhibition of miR-146 enhanced c-Jun expression in response to IL-1β (*n* = 4). Significant *p* values (*t*-test) from left to right are 0.005, 0.024, respectively.

MiR-146 targets TRAF6, IRAK1 and IRAK2 (Hou et al, 2009; Taganov et al, 2006), which are key adaptor molecules of the IL-1β signal transduction pathway. Several signalling pathways are activated downstream of TRAF6/IRAK1/2 including the NF-κB, p42/p44 MAPK and JNK/AP-1 pathways. We found that miR-146a over-expression inhibited the IL-1β-mediated induction of an NF-κB-dependent reporter in endothelial cells, while inhibiting miR-146 enhanced NF-κB activity in response to IL-1β treatment (Fig 5A). In addition, we assessed the activation of the p42/p44 MAPK pathway by measuring the levels of phosphorylated ERK (pERK). Levels of pERK were reduced in unstimulated miR-146a over-expressing cells, and the induction of pERK in response to IL-1β was also inhibited (Fig 5B, top). In contrast, pERK levels were enhanced in cells treated with miR-146 inhibitor (Fig 5B, bottom). EGR transcription factors are induced downstream of MEK (MAPKK) in the p42/p44 MAPK pathway (Saleem et al, 1995). We assessed the expression of *EGR-1* and *EGR-3* in response to IL-1β treatment and found that *EGR-1* and *EGR-3* were potently induced after only 1 h of IL-1β, and that *EGR-3* was induced to a greater extent than *EGR-1* (Fig 5C). Consistent with the reduced levels of pERK, we found that miR-146a over-expression inhibited the activation of *EGR-1* and *EGR-3* mRNA in response to IL-1β, while miR-146 inhibitors enhanced the induction of *EGR-3* mRNA (Fig 5D). Interestingly, we found that *EGR-3* was a predicted target of miR-146 (Fig 5E). To determine whether miR-146 could directly repress *EGR-3* we performed luciferase assays in which a region of the *EGR-3* 3′ UTR or a concatemer of the predicted miR-146 binding site, were inserted downstream of the luciferase open reading frame (ORF). As a control, we assessed luciferase activity of constructs bearing the *TRAF6* 3′ UTR. While *TRAF6* luciferase reporters were highly repressed in response to miR-146a over-expression (Fig 5E), *EGR-3* 3′ UTR (Supporting Information Fig S3) or concatemer constructs (Fig 5E), were refractory to miR-146-mediated repression. This suggests that miR-146 does not directly target *EGR-3*, but that it instead represses activation of EGR proteins via inhibition of upstream signal components (*i.e*., TRAF6/IRAK1/2). Finally, the activation of the AP-1 pathway also appeared to be modestly inhibited by miR-146 since the IL-1β-mediated induction of *c-Fos* was reduced in cells over-expressing miR-146a, while the induction of *c-Jun* was enhanced when miR-146 was inhibited (Fig 5F).

### EGR proteins control the transcription of the miR-146a/b genes

**Figure 6:**
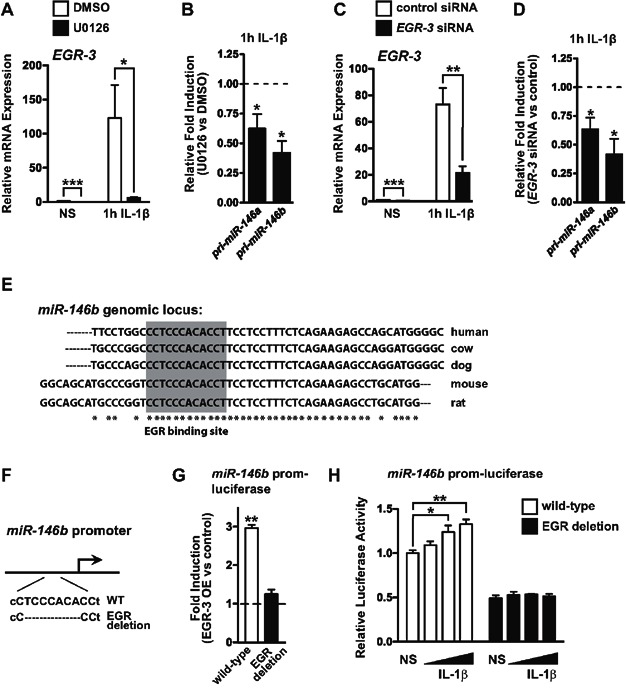
The MAPK/EGR pathway regulates the transcription of *miR-146a* and *miR-146b* Treatment of endothelial cells with the MEK inhibitor, U0126, inhibited the basal expression (*t*-test, *p* = 0.0003) and IL-1β-dependent induction (*t*-test, *p* = 0.037) of *EGR-3* (*n* = 3).Induction of *pri-miR-146a* and *pri-miR-146b* by IL-1β was reduced in cells pre-treated with the MAP kinase inhibitor, U0126. Data represents the relative induction of *pri-miR-146a/b* in cells treated with U0126 compared to cells treated with DMSO (vehicle) (*n* = 4). *p* = 0.037 for *pri-miR-146a* and *p* = 0.010 for *pri-miR-146b* (*t*-test).*EGR-3* knock-down by siRNA transfection reduced the basal (*t*-test, *p* < 0.0001) and IL-1β-induced levels (*t*-test, *p* = 0.004) of *EGR-3* (*n* = 5).The induction of *pri-miR-146a* and *pri-miR-146b* was also reduced in *EGR-3* knock-down cells (*n* = 5). *p* = 0.023 for *pri-miR-146a* and *p* = 0.013 for *pri-miR-146b* (*t*-test).Schematic indicating a potential EGR binding site (shaded area) in the *miR-146b* promoter. Sequence comparison between various species is indicated. Asterisks indicate conserved nucleotides across all species.Schematic of deletion of the EGR binding site in the *miR-146b* promoter.A *miR-146b* promoter-luciferase reporter was responsive to EGR-3 over-expression (OE) in bovine aortic endothelial cells (BAEC) and mutation of a conserved EGR binding site abrogated this responsiveness. Data depicts the fold induction with EGR-3 OE compared to control. Shown is a representative experiment (*n* = 3 replicates). *p* = 0.0017 (*t*-test).A *miR-146b* promoter-luciferase reporter was modestly induced in response to IL-1β and this induction was not observed when the *EGR* site was mutated. IL-1β was added at concentrations of 10, 20 or 40 ng/mL. Shown is a representative experiment (*n* = 3 replicates). ANOVA, *p* = 0.011. * and ** indicate a significant difference between the indicated groups, *p* < 0.05, *p* < 0.01, respectively.

Our data suggests that miR-146a and miR-146b may participate in a negative feedback loop in endothelial cells to restrain endothelial inflammation. *MiR-146a* is known to be NF-κB-responsive, while *miR-146b* is not (Perry et al, 2009). We found that miR-146a can repress the NF-κB signalling pathway (Fig 5A), revealing a miR-146a/NF-κB negative regulatory loop that acts to restrain inflammation in endothelial cells. To test whether a miR-146-mediated negative feedback loop might also involve EGR proteins, we antagonised the EGR pathway to assess if this pathway regulates the transcription of *miR-146a/b*. Inhibition of the MAP kinase pathway with the MEK inhibitor, U0126, repressed the rapid induction of *EGR-3* following a 1 h treatment with IL-1β (Fig 6A) and inhibited the induction of *pri-miR-146a* and *pri-miR-146b* at the same early time-point (Fig 6B). Similarly, knock-down of *EGR-3* by siRNA (Fig 6C) inhibited the rapid transcriptional induction of both *pri-miR-146a* and *pri-miR-146b* in response to IL-1β (Fig 6D). To define the *cis* elements that mediate this effect, we examined evolutionarily conserved regions (ECRs) surrounding the *miR-146a* and *miR-146b* genes for conserved EGR binding sites. No conserved EGR sites were found in the ECRs surrounding the promoter of *miR-146a* (10 kb up- and down-stream of the transcriptional start site of *pri-miR-146a*), suggesting that the EGR site(s) that mediate induction of *miR-146a* transcription may act at a distance or act through a non-conserved or non-canonical EGR site. However, a conserved EGR site was identified in the *miR-146b* promoter (858–848 nucleotides upstream of the mature miR-146b sequence; chr.10:104,195,419–104,195–428, Fig 6E). The transcriptional start site of *pri-miR-146b* is ∼700 nucleotides upstream of the miR-146b mature sequence (Taganov et al, 2006). This would place this EGR site in the proximal promoter of *miR-146b*. Over-expression of EGR-3 resulted in robust induction of a *miR-146b* proximal promoter/reporter construct, and mutation of the conserved EGR binding site in the *miR-146b* promoter (Fig 6F) eliminated this induction (Fig 6G). Furthermore, the *miR-146b* promoter was moderately responsive to IL-1β stimulation, and this effect was completely abrogated when the *EGR* binding site was mutated (Fig 6H). Taken together these data suggest that activation of the MAP kinase/EGR pathway regulates the transcription of the *miR-146a/b* loci, and that miR-146 can in turn repress the MAPK/EGR pathway; thereby forming a negative feedback loop.

### MiR-146 targets the RNA-binding protein HuR to control endothelial activation

**Figure 7:**
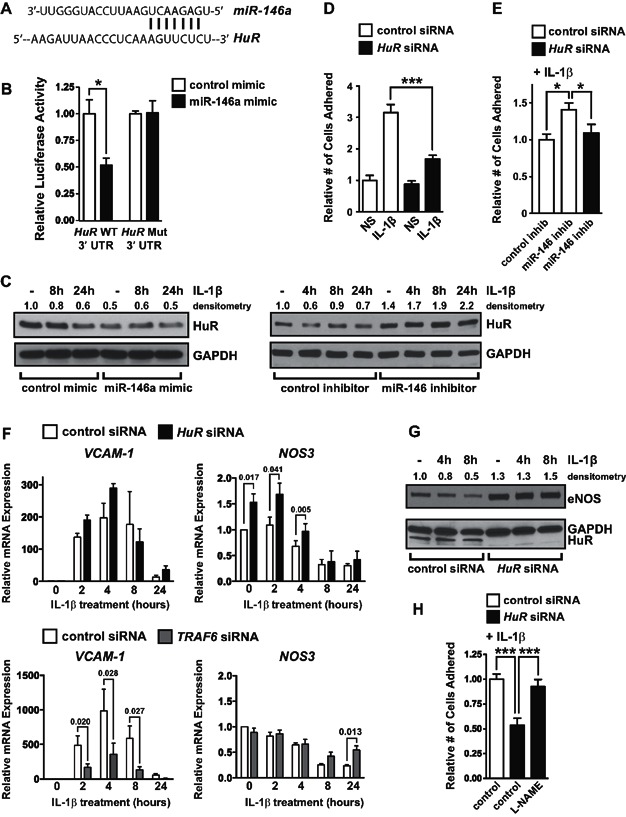
HuR, a novel miR-146 target, controls endothelial activation by regulating eNOS expression Schematic of a potential miR-146 binding site in the 3′ UTR of *HuR*.Luciferase assays utilizing wild-type (WT) or seed-mutated (Mut) *HuR* 3′ UTR sequences were performed in the presence of control or miR-146a mimic (mean ± SEM, *p* = 0.008, *t*-test, *n* = 4).HuR protein levels were quantified by Western blot in cells transfected with control or miR-146a mimic (left) or control or miR-146 inhibitor (right).The adhesion of THP-1 cells to vehicle or IL-1β treated cells transfected with control or *HuR* siRNAs revealed that HuR promotes endothelial activation. A representative experiment is shown (three replicate wells, three images per well). ANOVA, *p* < 0.0001. ***Indicates a significant decrease in THP-1 adhesion in IL-1β-treated *HuR* knock-down cells, *p* < 0.001.THP-1 adhesion assays were performed with endothelial cells transfected with control or miR-146 inhibitor and control or *HuR* siRNA. *HuR* knock-down reduced the elevated adhesion of THP-1 to endothelial cells transfected with miR-146 inhibitor. A representative experiment is shown (three replicate wells, three images per well). ANOVA = 0.016. *Indicates a significant difference between indicated groups, *p* < 0.05.Knock-down of *HuR* (above) or *TRAF6* (below) was performed and the induction of adhesion molecules (typified by *VCAM-1*) and eNOS (*NOS3*) was assessed by qRT-PCR. Expression of other inflammatory genes is indicated in Supporting Information Fig S9B. *HuR* knock-down did not reduce the induction of *VCAM-1*, in contrast to *TRAF6* knock-down, which strongly inhibited *VCAM-1* induction. However, *HuR* knock-down significantly elevated levels of *NOS3*. Shown is the mean ± SEM of three independent experiments. Significant *p* values (*t*-test) are indicated above.Levels of eNOS protein were elevated in *HuR* knock-down cells, and eNOS was not down-regulated in *HuR* knock-down cells treated with IL-1β.The nitric oxide inhibitor, l-NAME, negated the reduced THP-1 adhesion observed in *HuR* knock-down cells. A representative experiment is shown (three replicate wells, three images per well). ANOVA, *p* < 0.0001. ***Indicates a significant difference between groups, *p* < 0.001.

HuR was previously found to promote endothelial activation in response to LPS treatment of endothelial cells by facilitating NF-κB activation (Rhee et al, 2010). Interestingly, microRNA target prediction programs (Targetscan and Pictar) suggested that HuR might be a direct target of miR-146 (Fig 7, Fig. S4). We confirmed that luciferase constructs containing the *HuR* 3′ UTR could be repressed by miR-146a (Fig 7B), and also found that levels of HuR mRNA (Supporting Information Fig S5) and protein (Fig 7C) were suppressed or elevated when miR-146 was over-expressed or knocked-down in endothelial cells, respectively. To test the functional importance of HuR in IL-1β-mediated endothelial activation, we knocked down *HuR* and measured the adhesion of THP-1 cells to endothelial cells. *HuR* knock-down inhibited THP-1 adhesion to IL-1β treated endothelial cells (Fig 7D). Additionally, *HuR* knock-down also inhibited THP-1 adhesion to TNF-α treated cells (Supporting Information Fig S6), suggesting that HuR broadly facilitates endothelial activation. To assess the contribution of HuR to the enhanced adhesiveness of miR-146 inhibitor-treated endothelial cells, we knocked-down *HuR*, and were able to block the increase in THP-1 adhesion (Fig 7E). Interestingly, *VCAM-1*, *ICAM-1*, *SELE* and *MCP-1* contain AU-rich elements (AREs) in their 3′ UTRs (Supporting Information Fig S7). AREs can confer instability to transcripts, which is antagonized by HuR binding to these sites (Fan & Steitz, 1998). We therefore tested whether HuR could regulate the expression of these inflammatory genes. While *VCAM-1* and *MCP-1* were highly enriched in HuR immunoprecipitates from IL-1β-treated cells (Supporting Information Fig S8A), *HuR* knock-down failed to affect the induction of VCAM-1 or MCP-1 at the mRNA or protein level (Fig 7F, Supporting Information Figs S8B, C, and S9B), suggesting that they are not functional targets of HuR. Additionally, we found that NF-κB activity was not altered by *HuR* knock-down (Supporting Information Fig S8D), neither was the induction of *EGR-3* (Supporting Information Fig S9B). This was in contrast to knock-down of another miR-146 target, *TRAF6*, which decreased NF-κB activity (Supporting Information Fig S8D), the induction of adhesion molecules and *EGR* transcription factors (Fig 7F, Supporting Information Fig S9B). In contrast to the lack of regulation of VCAM-1/MCP-1 by HuR, eNOS mRNA and protein levels were elevated in *HuR* knock-down cells and eNOS was not down-regulated in response to IL-1β treatment of these cells (Fig 7F and G). Knock-down of *TRAF6* did not affect the basal levels of *NOS3* mRNA, but did inhibit the down-regulation of *NOS3* in response to IL-1β (Fig 7F). Finally, inhibition of nitric oxide activity by treatment with l-NAME negated the reduced adhesion in *HuR* knock-down cells (Fig 7H), suggesting that HuR regulates endothelial activation by modulation of NO activity. These results suggest that miR-146 targets TRAF6/IRAK1/2 and HuR, which cooperate to control endothelial activation through distinct pathways. While TRAF6/IRAK1/2 affects NF-κB transcriptional activity and the induction of leukocyte adhesion molecules and chemoattractants, HuR affects NO-dependent leukocyte adhesion.

### MiR-146a knock-out mice have an exaggerated acute vascular inflammatory response

**Figure 8:**
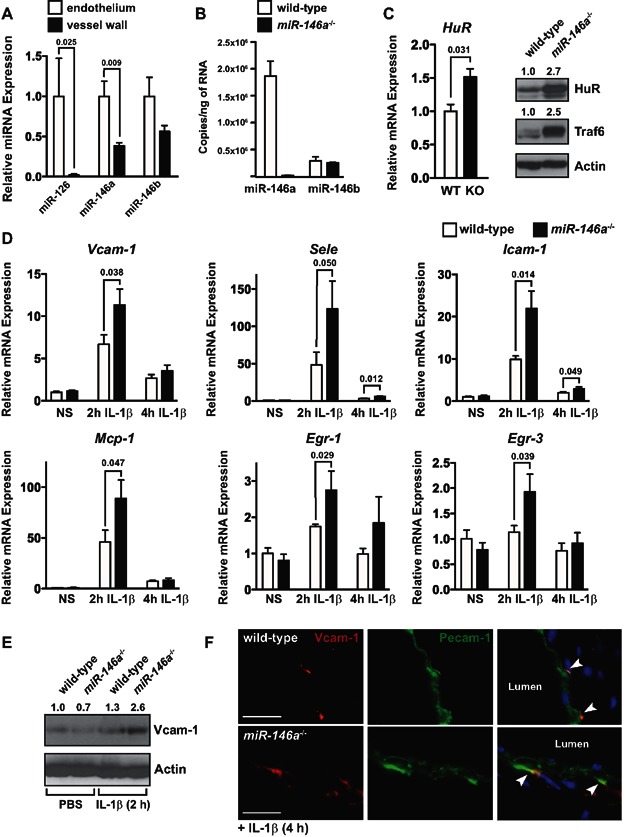
*miR-146a*^−/−^ mice demonstrate enhanced endothelial activation following IL-1β treatment Endothelial cells and cells in the vessel wall were isolated from the descending aorta of wild-type mice, and expression of miR-126 (as a control for endothelial cells) and miR-146a/b were measured by qRT-PCR. Expression was normalized to U6. MiR-146a was significantly enriched in the endothelium compared to the vessel wall (*n* = 4). Significant *p* values (*t*-test) are indicated above.Levels of miR-146a and miR-146b were quantified by qRT-PCR in hearts from wild-type and *miR-146a*^−/−^ mice (3–4 months of age, *n* = 3). Expression of miR-146a was >6-fold higher than miR-146b and miR-146b expression was not affected by loss of *miR-146a*.Expression of *HuR* mRNA was elevated in the hearts of *miR-146a*^−/−^ mice as assessed by qRT-PCR (left, *p* = 0.031, *t*-test, *n* = 3). Western blot revealed elevated levels of HuR and Traf6 (right).Wild-type and *miR-146a*^−/−^ mice (3–4 months of age, *n* = 4) were injected with PBS or 125 ng of IL-1β by tail vein injection and hearts were harvested after 2 or 4 h. Expression of inflammatory genes was assessed by qRT-PCR. While basal levels of these genes were unchanged in unstimulated mice (PBS injection), the induction of *Vcam-1*, *Icam-1*, *Sele*, *Mcp-1*, *Egr-1* and *Egr-3* was enhanced at 2 h in IL-1β treated mice, and *Sele* and *Icam-1* were still elevated at 4 h. Significant *p* values (*t*-test) are indicated above.Expression of Vcam-1 protein was elevated after a 2 h IL-1β treatment in *miR-146a*^−/−^ mice compared to wild-type mice.Localization of Vcam-1 expression was assessed by immunofluorescence, revealing an enhancement of Vcam-1 expression in the endothelium and in puncta adjacent to the endothelium of *miR-146a*^−/−^ mice treated with IL-1β for 4 h. Scale bars = 20 μm.

Assessment of miR-146a/b expression in blood vessels revealed that this microRNA family is enriched in the endothelium compared to cells in the vascular wall (Fig 8A). To assess the role of miR-146a in controlling endothelial activation *in vivo*, we utilized *miR-146a*^−/−^ mice (Boldin et al, 2011). *MiR-146a*^−/−^ mice on a C57/BL6 background are phenotypically normal at birth, but acquire chronic inflammation, including myeloproliferation in the spleen and bone marrow and develop enlarged spleens beginning around 5–6 months of age (Zhao et al, 2011). We therefore utilized young mice (3–4 months of age) for our experiments, since they do not appear to have an overt inflammatory phenotype. MiR-146a was expressed at much higher levels than miR-146b in the heart, and loss of *miR-146a* did not affect expression of miR-146b, suggesting that miR-146b is likely unable to compensate for loss of *miR-146a* (Fig 8B). Additionally, we assessed the expression of several other microRNAs that are known to modulate inflammatory signalling, and found that these were not appreciably altered in *miR-146a*^−/−^ mice (Supporting Information Fig S10). Similar to our findings using miR-146 inhibitors *in vitro*, we found that levels of HuR mRNA and protein were increased in the hearts of *miR-146a*^−/−^ mice (Fig 8C), suggesting that HuR is also a target of miR-146a *in vivo*. Levels of TRAF6 protein were also highly elevated (Fig 8C). To determine the role of miR-146a in the regulation of an acute vascular inflammatory response, wild-type and *miR-146a*^−/−^ mice were injected with PBS or IL-1β and the expression of several inflammatory genes were measured in harvested hearts. We found that the basal expression of these genes in PBS-injected mice was not altered in *miR-146a*^−/−^ mice compared to wild-type mice (Fig 8D). However, *miR-146a*^−/−^ mice had enhanced expression of *Vcam-1*, *Icam-1*, *Sele*, *Mcp-1*, *Egr-1* and *Egr-3* in response to a 2 h IL-1β treatment, and *Icam-1* and *Sele* remained significantly elevated at 4 h (Fig 8D). In contrast to markers of endothelial activation, levels of eNOS (*Nos3*) mRNA tended to be lower in *miR-146a*^−/−^ mice, although this difference did not reach statistical significance (Supporting Information Fig S11A). Levels of eNOS protein were also modestly reduced in *miR-146a*^−/−^ mice (Supporting Information Fig S11B). Enhanced induction of Vcam-1 protein in *miR-146a*^−/−^ mice was confirmed by Western blotting (Fig 8E) and immunofluorescence (Fig 8F). Vcam-1 protein was predominantly increased in the endothelium, although expression was also observed in regions immediately adjacent to the endothelium. Taken together, these data demonstrate that *miR-146a* restrains endothelial activation *in vivo*.

## DISCUSSION

Acute inflammation is essential for wound repair and for the innate immune response to invading pathogens. However, the intensity and duration of an acute inflammatory response must be tightly regulated, especially considering that inflammation has a detrimental effect on the function of the vasculature. For example, an excessive inflammatory response during sepsis results in organ failure and death due to profound and systemic increases in endothelial cell permeability (London et al, 2010), while chronic vascular inflammation drives the progression of atherosclerosis (Pober & Sessa, 2007). We demonstrate here that miR-146a and miR-146b act to restrain the intensity and duration of endothelial activation in response to pro-inflammatory cytokine stimulation. While miR-146a over-expression blunts endothelial activation and recruitment of leukocytes in response to IL-1β treatment, knock-down of miR-146a/b *in vitro* has the opposite effect. Importantly, *miR-146a*^−/−^ mice display enhanced induction of leukocyte adhesion molecules and chemokines in response to IL-1β treatment, demonstrating that miR-146a restrains vascular inflammation *in vivo*. We find that the anti-inflammatory activity of miR-146a/b is mediated by suppression of pro-inflammatory transcription factors (*i.e*., NF-κB, EGR-1/3, AP-1) as well as through modulation of post-transcriptional pro-inflammatory pathways (mediated by the targeting of HuR).

MiR-146a/b levels accumulate in the late stages of an inflammatory response, when other inflammatory genes such as *VCAM-1*, *ICAM-1* and *SELE* are being down-regulated (Fig 1), and miR-146a/b levels remain elevated for several days, even in the absence of pro-inflammatory cytokines (Fig 2). The initial transcription of *miR-146a* is mediated, to a large extent, by NF-κB (Taganov et al, 2006). We also identify a role for EGR-3 in the transcriptional regulation of both *miR-146a* and *miR-146b* (Fig 6). Since miR-146a/b repress activation of the NF-κB and EGR pathways (Fig 5), miR-146a/b induction in response to pro-inflammatory cytokines forms a negative feedback loop to control endothelial activation. Curiously, the NF-κB (Arenzana-Seisdedos et al, 1995) and EGR pathways (Fig 5C) are only transiently active following induction of inflammation, yet the transcription of *miR-146a/b* is maintained in the late stages of an inflammatory response (Fig 1D), and in the case of *miR-146b*, transcription is maintained even in the absence of cytokine (Fig 2C). The pathways that mediate this continued transcription are unknown. In addition, the mechanisms that control the delayed appearance of mature miR-146a/b during inflammation are also not known.

Considering the kinetics of miR-146 induction, we posit that miR-146 may play a role in the resolution of vascular inflammation and that the prolonged expression of miR-146 is a molecular marker of inflammatory ‘memory’. This is consistent with a recent report demonstrating that miR-146a is involved in the resolution of T-cell activation (Yang et al, 2012). In endothelial cells, elevated levels of miR-146a/b may promote cytokine desensitization, whereby an initial cytokine treatment blunts the intensity of a subsequent response to cytokine exposure (Pober et al, 1986, 1987). Others have observed that induction of miR-146a in monocytes following exposure to LPS promotes tolerance to this stimulus (Nahid et al, 2009; Nahid et al, 2011). Perhaps a similar mechanism involving miR-146a and/or miR-146b operates in endothelial cells to restrain inflammation in response to pro-inflammatory cytokines.

Such desensitization might serve to prevent chronic activation of inflammation in the vasculature, and we anticipate that miR-146 expression in the endothelium may therefore play a protective role against the development of atherosclerosis, a chronic inflammatory disease. While the expression of miR-146a and miR-146b is elevated in human atherosclerotic plaques (Raitoharju et al, 2011), the function of miR-146 in the progression of atherosclerosis is not known.

We find that miR-146 restrains vascular inflammation by repressing the NF-κB and EGR pathways, which play important roles in atherogenesis (Albrecht et al, 2010; Gareus et al, 2008; Harja et al, 2004). Additionally, miR-146a also targets TLR4 (Yang et al, 2011), which is expressed in several vascular and leukocyte cell types, and has been implicated in the etiology of atherosclerosis (den Dekker et al, 2010). We also identify HuR as a novel target of miR-146 and find that HuR acts to promote endothelial activation and leukocyte recruitment in response to IL-1β. A prior report demonstrated that HuR knock-down repressed endothelial activation *in vitro* in response to LPS. This was accompanied by a reduction in the activation of NF-κB and an elevation of eNOS mRNA (Rhee et al, 2010). While we also find that knock-down of *HuR* reduces the adhesion of monocytes to IL-1β treated endothelial cells (Fig 7D), HuR does not regulate NF-κB activity in IL-1β-treated cells (Supporting Information Fig S8D), nor does it regulate the induction of adhesion molecules (Fig 7F, Supporting Information Figs. S8B and S9B). Instead HuR represses the expression of eNOS and cells with reduced levels of HuR are not able to down-regulate eNOS expression in response to IL-1β treatment (Fig 7F and G). Importantly, eNOS down-regulation plays a key role in atherogenesis (Knowles et al, 2000; Oemar et al, 1998). In addition we show that inhibition of NO activity can rescue the reduced leukocyte adhesion observed in *HuR* knock-down cells (Fig 7H). While HuR does not directly bind to *NOS3* mRNA, it does bind to a known positive regulator of *NOS3* transcription (Lin et al, 2005), *KLF2* (Supporting Information Fig S12A), and knock-down of *HuR* results in elevated levels of *KLF2* (Supporting Information Fig S12B). Finally, we find that HuR protein levels are reduced at the late stages of endothelial activation (Fig 7C), suggesting that miR-146 up-regulation at this stage may repress HuR, thereby forming a negative feedback loop. MiR-146 therefore inhibits endothelial activation by coordinately repressing the induction of adhesion molecules (through targeting of TRAF6/IRAK1/2) and by promoting the expression of eNOS, an inhibitor of leukocyte adhesion (through targeting of HuR) (Supporting Information Fig S13).

From recent discoveries it appears that a microRNA network acts in endothelial cells to restrain inflammation (Fish & Cybulsky, 2012). For example, miR-10a levels are decreased in regions of the mouse aorta that are susceptible to the development of atherosclerosis (Fang et al, 2010). MiR-10a represses NF-κB activity by targeting MAP kinase kinase kinase 7 (MAP3K7, also known as TAK1) and β-transducin repeat-containing gene (β-TRC), which mediate IκB degradation (Fang et al, 2010). Additionally, TNF-α up-regulates miR-31 and miR-17-5p, which directly repress the adhesion molecule genes *SELE* and *ICAM1*, respectively (Suarez et al, 2010). More recently, miR-181b was found to repress the expression of importin-α3, which is required for the nuclear import of NF-κB proteins (Sun et al, 2012). Over-expression of miR-181b in the vasculature inhibits the expression of NF-κB-dependent genes and protects mice from sepsis (Sun et al, 2012). The existence of several microRNAs that converge on the NF-κB pathway suggests that tight control of this pathway is crucial for the maintenance of vascular homeostasis. Our findings have added miR-146a and miR-146b to this microRNA-mediated NF-κB regulatory network in the endothelium (Supporting Information Fig S13). In addition to regulating the NF-κB pathway, miR-146 also controls activation of the EGR and AP-1 pathways, which are known to drive inflammatory gene expression (De Caterina et al, 2010; Hajra et al, 2000), and miR-146 directly targets HuR, which promotes endothelial activation by antagonizing eNOS expression. This implies that miR-146 may have an even broader anti-inflammatory role than miR-10a, miR-31, miR-17-5p or miR-181b. Our findings suggest that strategies to enhance miR-146a or miR-146b in the vasculature may be therapeutically useful to dampen the endothelial response to inflammatory cytokines, and may potentially be used to shut off the reiterative inflammatory loop that drives atherogenesis or to quell the vascular damage associated with cytokine storm in the setting of sepsis.

## MATERIALS AND METHODS

### Reagents used

Recombinant human IL-1β and TNF-α were from Invitrogen, and were used at a concentration of 10 ng/mL. Mouse recombinant IL-1β was from R&D Systems. The MAP kinase inhibitor, UO126, was from Sigma–Aldrich and was dissolved in DMSO and used at a concentration of 10 μM. l-NAME was purchased from Sigma–Aldrich and was used at a concentration of 0.1 mM.

### Cell culture and treatments

Human umbilical vein endothelial cells (HUVEC) and media (Endothelial Cell Medium with 5% FBS and Endothelial Cell Growth Supplement) were purchased from ScienCell. Bovine aortic endothelial cells (BAEC) and media were purchased from Lonza. Cells were used at passages 3–8. HeLa-S3 and THP-1 cells were purchased from ATCC. HeLa cells were maintained in DMEM with 10% FBS and THP-1 cells were maintained in RPMI1640 with l-glutamine and 0.05 nM β-mercaptoethanol and 10% FBS.

### Monocyte adhesion assay

THP-1 cells were labelled with CellTracker™ Green (Invitrogen) immediately prior to the experiment. HUVEC were cultured to confluence in 12-well plates and were treated with IL-1β for 4 h. Labeled THP-1 cells (10^5^) were then added to each well for 90 min and unbound cells were removed by washing with PBS. For experiments using l-NAME, cells were treated with IL-1β and 0.1 mM l-NAME for 4 h, and THP-1 cells were allowed to adhere for 15 min. Adherent cells were fixed with 4% paraformaldehyde and imaged using a Leica Microsystems inverted fluorescent microscope (Model #DMIL) with an Olympus DP71 camera. Adherent THP-1 cells were quantified in three random fields of view per well using ImageJ. Triplicate wells were analysed for each experiment.

### Transfection

HUVEC were transfected at ∼50% confluency with control or miR-146a mimics (20 nM, Dharmacon), or non-targeting control, *EGR-3*, *HuR* or *TRAF6* siRNAs (Silencer Select s4544, 4390843, s4610 or s14389, respectively, 40 nM, Invitrogen) and analysed after 24–72 h. For inhibitor experiments, HUVEC were transfected at ∼90% confluency with control or miR-146a locked-nucleic acid (LNA) inhibitors (20 nM, Power Inhibitors, Exiqon) and analysed 48–72 h later. All HUVEC transfections were performed using RNAiMax (Invitrogen). HeLa cells were transfected with plasmids and microRNA mimics using Lipofectamine 2000 (Invitrogen; see Supporting Information for details).

### Bioinformatic analysis of *miR-146a* and *miR-146b* proximal promoter regions

The genomic regions surrounding the *miR-146a* and *miR-146b* transcriptional start sites were assessed for the presence of Evolutionary Conserved Regions (ECRs) using ECR Browser (http://ecrbrowser.dcode.org/), and rVista (http://rvista.dcode.org/) was used to identify conserved transcription factor binding sites.

### Luciferase assays and cloning

See Supporting Information for details.

### Gene expression analysis

RNA was isolated using Trizol (Invitrogen), reverse transcribed using the High-Capacity cDNA Reverse Transcription kit (Applied Biosystems), and quantitative reverse-transcriptase PCR (qRT-PCR) was performed as described previously (Fish et al, 2010). For analysis of *pri-miR-146a* and *pri-miR-146b*, RNA was treated with DNase I (Ambion) to remove traces of genomic DNA. Real-time PCR was conducted in triplicate using a Roche Lightcycler 480® with Roche 480 Probes Master Mix or LC 480 SYBR Green I Master (Roche) for Taqman® and Sybr green chemistries, respectively. Data was normalized to Tata box binding protein (TBP) or glyceraldehyde 3-phosphate dehydrogenase (GAPDH) using the Delta-Delta *C*_t_ method. The primers used are indicated in Supporting Information Table SI.

MiR-146a and U6 were reverse-transcribed using the Taqman® MicroRNA Reverse Transcription kit (Applied Biosystems) and analysed using Taqman Primer sets (Applied Biosystems). The miR-146a primer set did not cross react with miR-146b (<1% cross reactivity). Since the miR-146b primer set from Applied Biosystems cross-reacted with miR-146a, we used the miScript system (Qiagen) for analysis of miR-146b. MiScript primers for miR-146b only partially cross-reacted with miR-146a (<20% cross reactivity). To quantify the number of copies of miR-146a and miR-146b, comparison was made to a standard curve generated by reverse transcribing a known amount of miR-146a or miR-146b mimic (Dharmacon). The MiScript system was also used for the analysis of other microRNAs (miR-10a, miR-17, miR-31, miR-155 and miR-181b) in wild-type and *miR-146a*^−/−^ hearts. Expression was normalized to miR-126 in these experiments.

### HuR immunoprecipitation

HUVEC were harvested and lysed in RIPA buffer (Santa Cruz) containing protease inhibitors and 100 U/mL RNAse OUT (Invitrogen). Protein–RNA complexes were isolated from 1.75 mg of total clarified protein with 5 μg of either HuR antibody (Santa Cruz, G-8) or V5 antibody (Invitrogen) using 60 μL protein A/G beads (Santa Cruz) by rotation at 4°C for 4 h. Beads were washed 3× in RIPA buffer and resuspended in 1 mL Trizol (Invitrogen), followed by RNA isolation.

### Western blotting

Western blotting was performed as described (Fish et al, 2008). For analysis of pERK, HUVEC were serum starved overnight (in basal medium containing 0.1% FBS) prior to stimulation with IL-1β (20 ng/mL). The following antibodies were used: phospho-ERK (p42/44^Thr202/Tyr204^, Cell Signaling, 9101), ERK2 (Santa Cruz, C-14), E-Selectin (Santa Cruz, H-300), ICAM-1 (Santa Cruz, G-5), TRAF6 (Santa Cruz, D-10), eNOS [Santa Cruz, C-20, generously provided by P. Marsden (University of Toronto)], VCAM-1 (for human samples; Santa Cruz, E-10), Vcam-1 (for mouse samples; R&D Systems, AF643), HuR (for human samples; Santa Cruz, G-8), HuR (for mouse samples; Santa Cruz, 3A2), GAPDH (Santa Cruz, 0411), Actin (Sigma, A2066) and Vinculin (Santa Cruz, H-300). HRP-conjugated secondary antibodies were from Cell Signaling or Santa Cruz, and blots were developed using SuperSignal West Pico Chemiluminescence Substrate (Pierce).

**The paper explained**

PROBLEM:

Inflammation plays a vital role in acute and chronic diseases of the vasculature, including sepsis and atherosclerosis, respectively. Therapies that directly repress vascular inflammation are expected to impede the development of these diseases. However, current therapies are not able to specifically suppress inflammatory signalling in the vasculature.

RESULTS:

We find that the miR-146 microRNA family is induced by pro-inflammatory cytokines and acts to inhibit vascular inflammation by repressing the expression of leukocyte adhesion molecules on the surface of endothelial cells, a process known as endothelial activation. MiR-146 accomplishes this by repressing both transcriptional and post-transcriptional pro-inflammatory pathways in endothelial cells.

IMPACT:

Our findings suggest that therapies that augment miR-146 expression in the vascular endothelium may be protective against the development of inflammatory vascular diseases. As approaches for enhancing microRNA expression *in vivo* continue to improve, it may be feasible to target vascular inflammation by modulating the expression of miR-146 in the endothelium. Additionally, it is possible that alterations in the levels of miR-146 may predispose individuals to the development of vascular inflammatory diseases.

### Enzyme-linked immunosorbent assay (ELISA)

MCP-1 protein was quantified in supernatants using a Quantikine ELISA kit from R&D Systems, according to the manufacturer's recommendations.

### Mouse experiments

All animal protocols were approved by the Animal Care Committee at the University Health Network (Toronto). Adult (3–4 months) wild-type and *miR-146a*^−/−^ mice (on a C57/BL6 background) were injected with 100 μL of PBS or 125 ng of recombinant mouse IL-1β (in PBS) by intravascular injection. Hearts (including a portion of the ascending aorta) were harvested at 2 h or 4 h post-injection and processed for RNA or protein analysis. For analysis of microRNA expression in the endothelium, endothelial cells were isolated from the vessel wall using a modified protocol (Jongstra-Bilen et al, 2006). Briefly, descending thoracic aortae were dissected, adipose tissue was removed, and aortae were pinned en face in ice-cold PBS containing 1 mM aurintricarboxylic acid (Sigma). Tissues were treated with 5U DNase I (Fermentas) and Liberase TM (1:100 in Ca^2+^/Mg^2+^-containing PBS, Roche) for 8 min at 37°C. Intimal cells were visualized by overlaying 0.1 μM fluoresbrite polystyrene microspheres (Polysciences). Intimal cells were scraped gently with a 30G needle and harvested directly into RNA extraction buffer (RNAqueous-micro kit, Invitrogen). Endothelium-depleted vessel wall tissue was homogenized in RNA extraction buffer.

### Immunostaining

Cryosections were stained as described (Delgado-Olguin et al, 2012). Primary antibodies were: FITC-Pecam-1 (1:200) (BD Biosciences) and Vcam-1 (1:100) (Proteintech). Vcam-1 was detected by incubation with Alexa Fluor 647 Goat Anti-Rabbit (Invitrogen). Sections were imaged using an Eclipse Ni-U Nikon microscope and processed using NIS-Elements Imaging Software.

### Statistical analysis

Unless otherwise indicated, data represent the mean of at least three independent experiments and error bars represent the standard error of the mean. Pair-wise comparisons were made using a Student's *t*-test. Comparison of three or more groups was performed using a 1-way analysis of variance (ANOVA) with Newman–Keuls *post hoc* test. A *p*-value of 0.05 or less was considered to be statistically significant. In all figures *, ** and *** represent a *p*-value of ≤0.05, ≤0.01 and ≤0.001, respectively.

## Supplementary material

As a service to our authors and readers, this journal provides supporting information supplied by the authors. Such materials are peer reviewed and may be re-organized for online delivery, but are not copy-edited or typeset. Technical support issues arising from supporting information (other than missing files) should be addressed to the authors.

**Table SI.** Primers used for qRT-PCR.

**Figure S1.** TNF-α induces miR-146a and miR-146b expression.

**Figure S2.** Over-expression of miR-146a inhibits monocyte adhesion to IL-1β-treated bovine aortic endothelial cells (BAEC).

**Figure S3.** miR-146a does not directly regulate *EGR-3*.

**Figure S4.** A potential miR-146 binding site in *HuR* is highly conserved across species.

**Figure S5.** miR-146 controls the expression of *HuR* mRNA.

**Figure S6.** *HuR* knock-down represses THP-1 adhesion to TNF-α-treated endothelial cells.

**Figure S7.** Predicted AU-rich elements (AREs) in the 3′ UTRs of genes involved in endothelial activation.

**Figure S8.** HuR binds to *VCAM-1* and *MCP-1* mRNA but does not regulate the induction of these genes by IL-1β.

**Figure S9.** The miR-146 targets, *HuR* and *TRAF6*, have divergent effects on the induction of inflammatory genes.

**Figure S10.** MicroRNAs previously implicated in regulating inflammation are not appreciably altered in *miR-146a−/−* mice.

**Figure S11.** Expression of eNOS is modestly decreased in *miR-146a−/−* mice.

**Figure S12.** *KLF2* mRNA is bound by HuR and knock-down of *HuR* leads to increased levels of *KLF2* transcripts.

**Figure S13.** Schematic of a miR-146 feedback loop that controls endothelial activation.
