# Supplementary material for: MicroRNA-146 represses endothelial activation by inhibiting pro-inflammatory pathways
Source: EMBO Mol Med. 2013 Jun 3;5(7):949–66. doi: 10.1002/emmm.201202318 (PMC3721471; doi:10.1002/emmm.201202318)
Supplement: Supplementary file 1 [file emmm0005-0949-SD1.pdf]

MicroRNA-146 represses endothelial activation by inhibiting pro-inflammatory pathways

Henry S. Cheng, Nirojini Sivachandran, Andrew Lau, Emilie Boudreau, Jimmy L. Zhao, David Baltimore, Paul Delgado-Olguin, Myron I. Cybulsky and Jason E. Fish

Corresponding author: Jason Fish, University Health Network

Review timeline:

Submission date:	01 December 2012
Editorial Decision:	30 January 2013
Revision received:	10 April 2013
Editorial Decision:	22 April 2013
Revision received:	26 April 2013
Accepted:	29 April 2013

Transaction Report:

(Note: With the exception of the correction of typographical or spelling errors that could be a source of ambiguity, letters and reports are not edited. The original formatting of letters and referee reports may not be reflected in this compilation.)

Editor: Roberto Buccione

1st Editorial Decision 30 January 2013

Thank you for the submission of your manuscript to EMBO Molecular Medicine. We have now heard back from the three Reviewers whom we asked to evaluate your manuscript.

You will see that all three Reviewers are supportive of your work and underline its considerable interest. However, some significant concerns were raised that require your action. I feel it unnecessary to report each point in detail here as they are clearly stated by the Reviewers, but I will mention a few salient items.

Reviewer 1's enthusiasm is somewhat dampened by the fact that novelty is reduced by previous work on miR-146, while acknowledging the interest of endothelial cell angle. S/he also notes that additional experimental support is required to consolidate the mechanistic aspects. Reviewer 1 thus suggests a number of experimental approaches (listed in detail) that would significantly increase the impact and overall significance of the manuscript. I agree, especially since conceptual and factual novelty is an important criterion for publication in EMBO Molecular Medicine.

Reviewer 2, while generally supportive, also notes that previously published work does detract from novelty.

Reviewer 3 points to a number of issues, which partially coincide with Reviewer 1's concerns. For example Reviewer 3 is also concerned about which cells within the heart mediate the effects of IL-1b and requires additional mechanistic definition. S/he would also like you to consider a longer time point after IL-1beta administration to clarify the kinetics of inflammatory gene and miR146 expression. Reviewer 3 also mentions a few other issues that require your attention. Finally, I also

agree that the manuscript would benefit from a shortened Introduction section.

While publication of the paper cannot be considered at this stage, we would welcome a suitably revised submission, with the understanding that the Reviewers' concerns must be fully addressed with additional experimental data where appropriate.

Please note that it is EMBO Molecular Medicine policy to allow a single round of revision only and that, therefore, acceptance or rejection of the manuscript will depend on the completeness of your responses included in the next, final version of the manuscript.

I look forward to receiving your revised manuscript as soon as possible.

***** Reviewer's comments *****

Referee #1 (General Remarks):

This is a nicely written paper by Cheng et al. that examines the effects of miR-146, a known inflammatory-responsive microRNA, using various gain- and loss-of-function approaches in endothelial cells. They provide data to suggest that overexpression of miR-146a inhibits endothelial activation, while inhibition increases endothelial inflammatory markers. Consistent with other reports for this microRNA in leukocytes, miR-146 also inhibits both NF-kB and MAP Kinase pathways in endothelial cells. The authors reveal a new mechanistic insight that miR-146 inhibits HuR, an RNA binding protein implicated in pro-inflammatory gene regulation. In addition, they find that EGR3 induced the miR-146 promoter as part of a negative feedback loop in endothelial cells. Finally, they explore the expression of a cassette of inflammatory markers in heart tissue from miR-146-/- mice and show that they are increased.

Several previous reports (their refs: Bhaumik et al, 2008; Hou et al, 2009; Nahid et al, 2009; Taganov et al, 2006) have identified an important role for miR-146 in regulating a negative feedback loop in response to inflammatory signals. While these data provide potentially important information for a role of miR-146 in endothelial cells, some of the novelty is, in part, reduced in this paper despite the focus on endothelial cells. To strengthen a potential distinctive role for miR-146 in endothelial cells, several questions require attention. Mechanistic concerns are raised for whether TRAF6/IRAK1/2/ vs. HuR mediates miR-146's effects on expression of inflammatory markers in both endothelial cells in vitro and in the miR-146-/- mice in vivo. The potential role of EGR3 inducing miR-146 in response to IL-1b is provocative; however, it is not complete. Does mutation of the EGR site completely block IL-1b activation of miR-146 reporter? What is the kinetics of EGR3 induction in response to IL-1b compared to pri-miR-146 and mature miR-146-does EGR3 precede the miR or coincide with its expression? Finally, it is unclear from the miR-146-/- mice studies whether the effects of IL-1b stimulation are mediated by endothelial cells vs. other cell types within the heart tissue. Overall, these findings are quite relevant to the field of endothelial biology and have implications for inflammatory endothelial responses in various diseases. However, at this time, there are a number of methodological concerns and interpretation issues that should be further substantiated as detailed below to help explain and/or interpret their observations.

Major Concerns:

1) Figure 3/4 -mRNA expression of other relevant markers SELE, ICAM-1, MCP-1, NOS-3, and TF is shown to be reduced by miR-146a and induced by miR-146a inhibition. These should be verified on Western analyses in a similar manner as VCAM-1.
2) Currently the role of miR-146 is overstated in the paper in both the Results and Discussion as the authors highlight a role for this miR-146 in endothelial adhesion molecules, thrombogenicity (TF), and dysfunction(NOS3). Functional data is only shown for adhesion. Please either verify effects on TF-dependent clotting and NOS3-dependent endothelial function, or tone down such statements, accordingly.
3) Figure 5 - please show EGR3 kinetics on both the mRNA and protein levels in response to IL-1b and compare to pri-miR-146a and miR-146a. Does EGR3 expression precede induction of miR-146?
4) Figure 6F-does mutation of the EGR site completely block IL-1b responsiveness on the miR-146 promoter?
5) Figure 7-does overexpression of miR-146a inhibit HuR protein expression? This is a critical point

for this new target. In addition, does IL-1b regulate HuR expression? What is the HuR kinetics on both the mRNA and protein levels in response to IL-1b? Is the 3?UTR of HuR conserved between human and mouse?
6) A previous study showed that HuR knockdown in HUVECs inhibits NF-kB phosphorylation and DNA-binding to the VCAM-1 promoter in response to LPS (Rhee WJ et al, PNAS 2010). Given that HuR is proposed to be a direct target of miR-146, does miR-146 regulate NF-kB phosphorylation or NF-kB DNA-binding in HUVECs and is this dependent on HuR? To what extent does miR-146 overexpression reduce TRAF6 expression at the protein level?
7) In endothelial cells, how does miR-146a mediate inhibition of inflammatory markers? Is it mediated through TRAF6/IRAK1/2/ vs. HuR in vitro? For example, siRNA knockdown studies in the presence of miR-146 mimics on expression or endothelial function (adhesion, e.g. similar to Fig.7F) may provide insights.
8) Figure 8 - it is unclear from the miR-146-/- mice studies whether the effects of IL-1b stimulation are mediated by endothelial cells vs. other cell types within the heart tissue. Immunohistochemistry co-localization studies of these markers with endothelial cells and/or isolation of heart endothelial cells would be informative.
9) Figure 8-What is the relative role of TRAF6/IRAK1/2 vs HuR in expression of markers in the miR-146-/- mice in vivo in response to IL-1b?

Minor:
1) General-do other proinflammatory stimuli besides Il-1b similarly induce miR-146 and HuR responsiveness?
2) In Figure3 and 4, does miR-146 dampen EC activation in HAECs in response to IL-1b?
3) Is expression of other relevant microRNAs involved in endothelial cells and inflammation (ie. miR-10a, miR-31,etc) altered in the miR-146-/- mice in response to IL-1b? This is relevant as microRNA-dependent effects on other microRNAs have emerged as an important mechanism.

Referee #2 (Comments on Novelty/Model System):

Well conducted cell and molecular analyses based upon reliable approaches used to manipulate inflammatory pathways in cultured endothelial cells and accompanied by nominal in vivo supportive data in a murine miRNA KO model.
Novelty:- The novelty is slightly reduced because of the Baltimore group's earlier identification of miR146 (and other miRs) in checking inflammation in monocytes via negative feedback (Taganov et al 2006). Nevertheless, the endothelium is a key gatekeeper of vascular homeostasis and the in vivo evidence here suggests that mir146 may play a more dominant role in this cell type.
Medical impact:- Relevance to the regulation of chronic inflammatory diseases in which the endothelium is a critical early facilitator is high; - includes atherogenesis as well as devastating systemic inflammations such as sepsis.

Referee #2 (General Remarks):

Cheng et al investigated mechanisms and effects of miR-146 induction following cytokine activation of inflammation in vascular endothelium. MiR146a/b were previously shown in monocytes to be part of a complex control system to keep innate immunity and inflammation in check through a negative feedback regulation loop (Taganov et al 2006) ; however, similar mechanisms have not been reported in inflammation in the endothelium (although enriched miR146 in endothelial cells derived from differentiating ESCs was reported earlier by this group). The work therefore is a logical and important conceptual extension of the monocyte studies and has uncovered both similarities and important differences in the contribution of miR146 in the delayed repression of endothelial inflammation. The studies are well conducted, the manuscript is clearly written, and the results convincing. Of particular note are the timing of induction of miR146, its sustained effects in checking inflammatory markers, the pathways involved and in particular the identification of the RNA binding protein HuR as a target. The miR146a -/- mouse studies add in vivo credibility that mir146 is a significant contributor to the checks and balances of arterial inflammation mediated through the endothelium. Both the introduction and discussion are thorough in defining the context of the result within a complex network of control of inflammation, including the roles of other miRs and regulators. Furthermore, the manuscript is a pleasure to read.

Referee #3 (General Remarks):

The authors have explored the role of miR146a on endothelial inflammatory activation. By using sound in vitro experiments, they found that IL- -induced miR146a/b exerts anti-inflammatory activity, as assessed by diminished expression of VCAM-1, ICAM-1, Eselectin and MCP-1, by repressing the activity of the pro-inflammatory transcription factors, NF-kB, EGR-1/3 and AP-1. They also showed that miR146a modulate post-transcriptional pathways mediated by the targeting of HuR. Finally, they confirmed their in vitro findings in miR146a-/- mice.

Major comments.

1. One the major in vitro finding of this manuscript is the inverse kinetics of inflammatory gene and miR146a/b expression after IL- activation, which accounts for the rapid down-regulation of adhesion molecule genes (figure 1A,B). Therefore one expects VCAM-1, ICAM-1, Eselectin and MCP-1expression to rapidly decrease after IL- injection in WT mice, but sustainably expressed in miR146a-/- mice. As the authors used only one time point (2h), this important piece of information cannot be verified. Analysis of inflammatory gene expression at a longer time point after IL- injection must be considered.
2. NOS3 expression is reduced by miR146-inhibitor treatment in vitro. Does this translate in vivo? What is the level of NOS3 expression in miR146a-/- mice in basal condition and after IL- injection.
3. Fig 2B. 24h after IL-1b removal, miR146a/b expression seems to be increased compared to T0. Is that statistically significant? If yes, any explanation?
4. Fig 3A. Over-expression of miR146a increased NOS3 mRNA expression, which contrasts with the down-regulation of adhesion molecule genes. Even though the explanation might not be easy, the authors should discuss this finding in the Discussion section.
5. Fig 8A. miR146a/b expression was analysed in the heart. Is miR146a/b expressed by cardiomyocytes? Expression in the aorta should be reported. What about the expression in low- and high-flow region (curvature vs descending aorta)?
6. Fig 8 C. HuR mRNA expression id reported. What about HuR protein expression?
7. Was NOS3 expression altered in miR146a-/- mice ? How was its expression modulated by IL- injection in WT and miR146a-/- mice? Same for EGR3.

Minor comment.

The Introduction is much too long.

1st Revision - authors' response 10 April 2013

Response to Reviewers' Comments:

We thank the Reviewers for their thoughtful and constructive review of our manuscript. We have endeavoured to revise our manuscript according to the comments we have received. These valuable suggestions for revisions have resulted, we believe, in an improved submission. A detailed point-by-point response is included below, with the Reviewers' comments (included in their entirety) indicated in italics.

Referee #1 (General Remarks):

This is a nicely written paper by Cheng et al. that examines the effects of miR-146, a known inflammatory-responsive microRNA, using various gain- and loss-of-function approaches in endothelial cells. They provide data to suggest that overexpression of miR-146a inhibits endothelial activation, while inhibition increases endothelial inflammatory markers. Consistent with other

reports for this microRNA in leukocytes, miR-146 also inhibits both NF-kB and MAP Kinase pathways in endothelial cells. The authors reveal a new mechanistic insight that miR-146 inhibits HuR, an RNA binding protein implicated in pro-inflammatory gene regulation. In addition, they find that EGR3 induced the miR-146 promoter as part of a negative feedback loop in endothelial cells. Finally, they explore the expression of a cassette of inflammatory markers in heart tissue from miR-146-/- mice and show that they are increased.

Several previous reports (their refs: Bhaumik et al, 2008; Hou et al, 2009; Nahid et al, 2009; Taganov et al, 2006) have identified an important role for miR-146 in regulating a negative feedback loop in response to inflammatory signals. While these data provide potentially important information for a role of miR-146 in endothelial cells, some of the novelty is, in part, reduced in this paper despite the focus on endothelial cells. To strengthen a potential distinctive role for miR-146 in endothelial cells, several questions require attention. Mechanistic concerns are raised for whether TRAF6/IRAK1/2/ vs. HuR mediates miR-146's effects on expression of inflammatory markers in both endothelial cells in vitro and in the miR-146-/- mice in vivo. The potential role of EGR3 inducing miR-146 in response to IL-1b is provocative; however, it is not complete. Does mutation of the EGR site completely block IL-1b activation of miR-146 reporter? What is the kinetics of EGR3 induction in response to IL-1b compared to pri-miR-146 and mature miR-146-does EGR3 precede the miR or coincide with its expression? Finally, it is unclear from the miR-146-/- mice studies whether the effects of IL-1b stimulation are mediated by endothelial cells vs. other cell types within the heart tissue. Overall, these findings are quite relevant to the field of endothelial biology and have implications for inflammatory endothelial responses in various diseases. However, at this time, there are a number of methodological concerns and interpretation issues that should be further substantiated as detailed below to help explain and/or interpret their observations.

We thank the Reviewer for their positive comments regarding our manuscript and its relevance to the field of endothelial biology. We agree that the role of miR-146 in controlling NF-κB activation is well-established in leukocytes, although it has not been explored in endothelial cells. As suggested by the Reviewer we have further investigated the role of the miR-146 targets, TRAF6 and HuR, in endothelial activation, and have surprisingly found that they play non-overlapping, but complementary roles in controlling endothelial activation. While knock-down of the adaptor molecule, TRAF6, blunted the activation of the NF-κB (Supporting Information Fig. S8D) and EGR (Fig. S9B) pathways, and the induction of endothelial adhesion molecules (Fig. 7F and Fig. S9B), knock-down of HuR had no effect on the IL-1β-mediated activation of NF-κB (Fig. S8D) or EGR (Fig. S9B), or the induction of adhesion molecules (Fig. 7F and Fig. S9B). Instead, suppression of HuR lead to elevated levels of eNOS (Fig. 7F,G). eNOS-derived NO is known to inhibit leukocyte adhesion, and importantly we show that blocking NO production rescued the anti-inflammatory effects of HuR knock-down (Fig. 7H). Interestingly, eNOS was also modestly reduced in *miR-146a$^{-/-}$* mice (Fig. S11A,B). In addition, we have further investigated the role of EGR-3 in activating the *miR-146b* promoter (Fig. 6H) and have spatially defined the induction of Vcam-1 protein *in vivo* (Fig. 8F). These findings are described in detail below.

Major Concerns:

1) Figure 3/4 -mRNA expression of other relevant markers SELE, ICAM-1, MCP-1, NOS-3, and TF is shown to be reduced by miR-146a and induced by miR-146a inhibition. These should be verified on Western analyses in a similar manner as VCAM-1.

We have added additional western blots to demonstrate altered expression of eNOS, E-Selectin and ICAM-1 protein in response to over-expression (Fig. 3C,D) or inhibition (Fig. 4D,E) of miR-146. We have removed the data on Tissue Factor (see response to comment #2 below). We have also included TRAF6 western blots (Fig. 3A, 4A, 8C) to confirm that the over-expression/inhibition of miR-146 resulted in the expected decrease/increase of a known target of miR-146, respectively (see response to comment #6 below).

2) Currently the role of miR-146 is overstated in the paper in both the Results and Discussion as the authors highlight a role for this miR-146 in endothelial adhesion molecules, thrombogenicity (TF), and dysfunction (NOS3). Functional data is only shown for adhesion. Please either verify effects on TF-dependent clotting and NOS3-dependent endothelial function, or tone down such statements, accordingly.

We thank the Reviewer for this valuable comment regarding the interpretation of our data. We agree that the original manuscript did not contain any functional data regarding thrombogenicity. As this was not the main focus of the paper, we have removed the data on Tissue Factor expression and have modified the Results/Discussion accordingly to focus the paper on endothelial activation. In regards to eNOS expression, we have provided new data that demonstrates that miR-146 regulates eNOS protein levels (Fig. 3D, 4D, Fig. S11B), and present data that suggests that this is mediated by miR-146-dependent regulation of HuR (Fig. 7G). Importantly, while nitric oxide (NO) is known to regulate vascular tone, it is also a potent inhibitor of leukocyte adhesion, and therefore endothelial activation. We have therefore emphasized this later function of NO, which complements our investigation of adhesion molecule induction. Additionally, we demonstrate that knock-down of HuR decreases monocyte adhesion to endothelial cells in part by elevating levels of eNOS and nitric oxide activity (Fig. Fig. 7G,H). The data on eNOS regulation adds to the focus of the paper, which is on endothelial activation and monocyte adhesion to the endothelium.

3) Figure 5 - please show EGR3 kinetics on both the mRNA and protein levels in response to IL-1b and compare to pri-miR-146a and miR-146a. Does EGR3 expression precede induction of miR-146?

Despite repeated attempts using two different antibodies we have been unable to successfully detect EGR-3 induction by western blot. We believe this is a technical issue, and that EGR-3 protein levels do indeed rapidly increase in response to IL-1β. We found that *EGR-3* mRNA was induced by several hundred-fold by 1 hour post-IL-1β treatment (Fig. 5C), a time-point where we also observed robust induction of *pri-miR-146a* and *pri-miR-146b* transcripts (Fig. 1D). Importantly, knock-down of *EGR-3* prevented the induction of *pri-miR-146a/b* (Fig. 6D) at very early time-points (i.e. 1 hour post-IL-1β treatment), suggesting that induction of *EGR-3* is upstream of *pri-miR-146a/b* induction. On page 11 of our revised manuscript we have emphasized that the knock-down of *EGR-3* or inhibition of MAP kinase, decreased transcription of *pri-miR-146a/b* at the 1 hour time-point.

4) Figure 6F-does mutation of the EGR site completely block IL-1b responsiveness on the miR-146 promoter?

We measured the IL-1β-mediated induction of the *miR-146b* promoter-luciferase construct in bovine aortic endothelial cells (BAEC) (Fig. 6H). We found that promoter activity was significantly induced in response to IL-1β. Importantly, mutation of the *EGR* site completely blocked the responsiveness to IL-1β. Importantly, we also provide evidence that EGR-3 regulates the expression of endogenous *miR-146b* (Fig. 6B,D).

5) Figure 7-does overexpression of miR-146a inhibit HuR protein expression? This is a critical point for this new target. In addition, does IL-1b regulate HuR expression? What is the HuR kinetics on both the mRNA and protein levels in response to IL-1b? Is the 3?UTR of HuR conserved between human and mouse?

We have included additional data in miR-146 over-expression/inhibition experiments that demonstrate that HuR mRNA/protein expression is highly regulated by miR-146 (Fig. 7C and Fig. S5). Our data also suggests that HuR protein expression is decreased in response to prolonged IL-1β treatment (i.e. 24 h, Fig. 7C). Since HuR is pro-inflammatory, this finding would be consistent with a negative feedback role of miR-146 induction in the later stages of the inflammatory response. Finally, we have included sequence comparison of the miR-146 binding site in the *HuR* 3' UTR in

human, mouse and rat (Fig. S4) that demonstrates that this region is 100% conserved across these species.

6) A previous study showed that HuR knockdown in HUVECs inhibits NF-kB phosphorylation and DNA-binding to the VCAM-1 promoter in response to LPS (Rhee WJ et al, PNAS 2010). Given that HuR is proposed to be a direct target of miR-146, does miR-146 regulate NF-kB phosphorylation or NF-kB DNA-binding in HUVECs and is this dependent on HuR? To what extent does miR-146 overexpression reduce TRAF6 expression at the protein level?

We have further examined the mechanisms implicated in the pro-inflammatory function of HuR. In contrast to the findings of Rhee *et al*, we did not find that HuR knock-down affected NF-κB activity (Fig. S8D) or the induction of NF-κB-regulated genes such as *VCAM-1, ICAM-1* and *MCP-1* (Fig. 7F, Fig. S9B) in response to IL-1β treatment. Interestingly, we found that *VCAM-1* and *MCP-1* mRNA can be bound by HuR protein (Fig. S8A), but knock-down of HuR did not affect the protein levels of these targets (Fig. S8B,C), suggesting that HuR does not affect the induction of adhesion molecules. However, similar to Rhee *et al* we found that HuR knock-down resulted in elevated levels of eNOS mRNA/protein (Fig. 7F,G). Additionally, we provide functional data showing that the decreased monocyte adhesion in HuR knock-down cells is mediated, at least in part, by elevated eNOS expression and nitric oxide activity (Fig. 7H). While *eNOS* mRNA was not a direct target of HuR (Fig. S8A), we provide evidence that HuR can bind and regulate the expression of *KLF2*, a potent regulator of *eNOS* transcription (Fig. S12A,B). In contrast to the effect of HuR on endothelial activation, we found that knock-down of the adaptor molecule, TRAF6, resulted in the expected reduction in NF-κB activity (Fig. S8D) and reduced induction of NF-κB-dependent adhesion molecules (Fig. 7F, Fig. S9B). Therefore we demonstrate that the targeting of adaptor molecules (i.e. TRAF6/IRAK1/2) and HuR by miR-146 elicits non-overlapping, but complementary effects on endothelial activation. While the targeting of TRAF6/IRAK1/2 affects NF-κB transcriptional activity and adhesion molecule expression, targeting of HuR affects endothelial activation via the regulation of eNOS and nitric oxide production. While we have not included data on NF-κB phosphorylation, it is clear from Fig. 5A that miR-146 has a major effect on NF-κB transcriptional activity. Finally, we have also included western blot analyses of TRAF6 expression in cells and mice with altered levels of miR-146 (Fig. 3A, 4A, 8C).

7) In endothelial cells, how does miR-146a mediate inhibition of inflammatory markers? Is it mediated through TRAF6/IRAK1/2/ vs. HuR in vitro? For example, siRNA knockdown studies in the presence of miR-146 mimics on expression or endothelial function (adhesion, e.g. similar to Fig.7F) may provide insights.

We have investigated this question in detail (see response to comment #6 above). Importantly, we find that the effect of HuR on monocyte adhesion is mediated, at least in part, by regulation of eNOS and nitric oxide activity (Fig. 7H). This is a novel finding that greatly adds to our understanding of the pathways that are regulated by miR-146 in endothelial cells. In contrast, targeting of TRAF6/IRAK1/2 appears to regulate the induction of inflammatory adhesion molecules. The targeting of these two targets (HuR and TRAF6/IRAK1/2) appears to have a cooperative effect on the regulation of endothelial activation.

8) Figure 8 - it is unclear from the miR-146-/- mice studies whether the effects of IL-1b stimulation are mediated by endothelial cells vs. other cell types within the heart tissue. Immunohistochemistry co-localization studies of these markers with endothelial cells and/or isolation of heart endothelial cells would be informative.

We have provided immunofluorescence data that demonstrates that Vcam-1 protein levels are increased in the endothelium, as well as in regions directly adjacent to the endothelium, in *miR-146a*$^{-/-}$ mice treated with IL-1β (Fig. 8F). This suggests that the induction of *Vcam-1* mRNA that we observe is due in large part to increases in expression within the endothelium. The enhancement in

adhesion molecules within the endothelium in *miR-146a*$^{-/-}$ mice is consistent with the enrichment of miR-146a/b that we observe in the endothelium compared to cells in the vessel wall (Fig. 8A). Since E-Selectin and Icam-1 are largely induced on the surface of activated endothelial cells (Weyrich *et al, J Leukoc Biol*, 1995), we anticipate that their induction is similarly increased in endothelial cells in *miR-146a*$^{-/-}$ mice.

9) Figure 8-What is the relative role of TRAF6/IRAK1/2 vs HuR in expression of markers in the miR-146-/- mice in vivo in response to IL-1b?

We found that both TRAF6 and HuR protein levels are elevated in *miR-146a*$^{-/-}$ mice (Fig. 8C). Since we found that the induction of adhesion molecules were enhanced in *miR-146a*$^{-/-}$ mice, this suggests that the TRAF6/IRAK1 arm of the miR-146-regulated pathway is altered in these mice. While we did not find a significant decrease in *eNOS* mRNA in *miR-146a*$^{-/-}$ mice, there was a trend towards decreased expression (Fig. S11A). We also found that eNOS protein levels were modestly decreased in *miR-146a*$^{-/-}$ mice (Fig. S11B). This suggests that the elevated levels of HuR in *miR-146a*$^{-/-}$ mice may be responsible for the altered expression of eNOS. Further experiments using *Traf6*, *Irak1* or *HuR* heterozygous mice could determine whether elevated levels of these proteins are responsible for the enhanced inflammation and endothelial activation in *miR-146a*$^{-/-}$ mice, but these experiments are beyond the scope of this manuscript.

Minor:

1) General-do other proinflammatory stimuli besides Il-1b similarly induce miR-146 and HuR responsiveness?

In Fig. S1A we show that another pro-inflammatory cytokine, TNF-α, also induces miR-146a/b expression, with similar kinetics. Additionally, we demonstrate that knock-down of HuR in endothelial cells also reduced the adhesion of monocytes in response to TNF-α stimulation (Fig. S6), suggesting that HuR may broadly promote endothelial activation.

2) In Figure3 and 4, does miR-146 dampen EC activation in HAECs in response to IL-1b?

Since all of our experiments were performed in HUVEC, a venous cell type, it is indeed important to demonstrate that similar effects of miR-146 are seen in arterial aortic endothelial cells. We have therefore over-expressed miR-146a in bovine aortic endothelial cells and observed a reduction in monocyte adhesion in response to IL-1β (Fig. S2). This suggests that miR-146 is likely to have similar anti-inflammatory properties in aortic endothelial cells.

3) Is expression of other relevant microRNAs involved in endothelial cells and inflammation (i.e. miR-10a, miR-31,etc) altered in the miR-146-/- mice in response to IL-1b? This is relevant as microRNA-dependent effects on other microRNAs have emerged as an important mechanism.

We have assessed the expression of several microRNAs in *miR-146a*$^{-/-}$ mice that are known to affect inflammatory signalling (i.e. miR-10a, miR-17, miR-31, miR-155 and miR-181b). We found no appreciable alterations in the expression of these microRNAs (Fig. S10). Since all our studies were using acute inflammatory models, we believe that measuring the basal levels of these microRNAs is informative. In future studies of chronic inflammation, the expression of these microRNAs after longer durations of inflammation will be important to assess, but this is beyond the scope of this manuscript.

Referee #2 (Comments on Novelty/Model System):

Well conducted cell and molecular analyses based upon reliable approaches used to manipulate inflammatory pathways in cultured endothelial cells and accompanied by nominal in vivo supportive data in a murine miRNA KO model.

Novelty:- The novelty is slightly reduced because of the Baltimore group's earlier identification of miR146 (and other miRs) in checking inflammation in monocytes via negative feedback (Taganov et al 2006). Nevertheless, the endothelium is a key gatekeeper of vascular homeostasis and the in vivo evidence here suggests that mir146 may play a more dominant role in this cell type.

Medical impact:- Relevance to the regulation of chronic inflammatory diseases in which the endothelium is a critical early facilitator is high; - includes atherogenesis as well as devastating systemic inflammations such as sepsis.

Referee #2 (General Remarks):

Cheng et al investigated mechanisms and effects of miR-146 induction following cytokine activation of inflammation in vascular endothelium. MiR146a/b were previously shown in monocytes to be part of a complex control system to keep innate immunity and inflammation in check through a negative feedback regulation loop (Taganov et al 2006) ; however, similar mechanisms have not been reported in inflammation in the endothelium (although enriched miR146 in endothelial cells derived from differentiating ESCs was reported earlier by this group). The work therefore is a logical and important conceptual extension of the monocyte studies and has uncovered both similarities and important differences in the contribution of miR146 in the delayed repression of endothelial inflammation. The studies are well conducted, the manuscript is clearly written, and the results convincing. Of particular note are the timing of induction of miR146, its sustained effects in checking inflammatory markers

, the pathways involved and in particular the identification of the RNA binding protein HuR as a target. The miR146a -/- mouse studies add in vivo credibility that mir146 is a significant contributor to the checks and balances of arterial inflammation mediated through the endothelium. Both the introduction and discussion are thorough in defining the context of the result within a complex network of control of inflammation, including the roles of other miRs and regulators. Furthermore, the manuscript is a pleasure to read.

We thank the Reviewer for their positive assessment of our manuscript, especially concerning the novelty and relevance of our findings to vascular inflammatory disease.

Referee #3 (General Remarks):

The authors have explored the role of miR146a on endothelial inflammatory activation. By using sound in vitro experiments, they found that ILb-induced miR146a/b exerts anti-inflammatory activity, as assessed by diminished expression of VCAM-1, ICAM-1, Eselectin and MCP-1, by repressing the activity of the pro-inflammatory transcription factors, NF-kB, EGR-1/3 and AP-1. They also showed that miR146a modulate post-transcriptional pathways mediated by the targeting of HuR. Finally, they confirmed their in vitro findings in miR146a-/- mice.

Major comments.

1. One the major in vitro finding of this manuscript is the inverse kinetics of inflammatory gene and miR146a/b expression after IL1b activation, which accounts for the rapid down-regulation of

adhesion molecule genes (figure 1A,B). Therefore one expects VCAM-1, ICAM-1, Eselectin and MCP-1 expression to rapidly decrease after IL1b injection in WT mice, but sustainably expressed in miR146a-/- mice. As the authors used only one time point (2h), this important piece of information cannot be verified. Analysis of inflammatory gene expression at a longer time point after IL1b injection must be considered.

We thank the Reviewer for this valuable suggestion. We have added an additional time-point (4 h) following IL-1β administration (Fig. 8D). We found that expression of adhesion molecules largely returned to baseline levels by this time-point. Importantly, expression of *E-Selectin* and *Icam-1* were significantly elevated in *miR-146a*$^{-/-}$ mice compared to wild-type mice at the 4 h time-point, and there was a trend towards increased *Vcam-1* and *Egr-1* expression as well. These *in vivo* experiments are suggestive that loss of *miR-146a* may prolong the expression of vascular inflammatory markers during an acute inflammatory response.

2. NOS3 expression is reduced by miR146-inhibitor treatment in vitro. Does this translate in vivo? What is the level of NOS3 expression in miR146a-/- mice in basal condition and after IL1b injection.

We have assessed the expression of eNOS mRNA/protein in wild-type and *miR-146a*$^{-/-}$ mice (Fig. S11). While not achieving statistical significance, there was a trend toward lower eNOS mRNA levels in unstimulated knock-out mice (Fig. S11A). This is consistent with our results *in vitro*. Additionally, eNOS protein levels were decreased in these mice under basal conditions (Fig. S11B). In response to IL-1β treatment, levels of eNOS mRNA decreased similarly in wild-type and knock-out mice (Fig. S11A). These findings suggest that regulation of eNOS expression may be regulated by miR-146 *in vivo*. In future experiments it will be informative to look at eNOS expression in *miR-146a*$^{-/-}$ mice in models of chronic inflammation, such as atherosclerosis, where eNOS is known to be highly down-regulated (Knowles *et al*, *J Clin Invest*, 2000; Oemar *et al*, *Circulation*, 1998). We would anticipate that the loss of eNOS expression would be enhanced and/or accelerated in *miR-146a*$^{-/-}$ mice in these models.

3. Fig 2B. 24h after IL-1b removal, miR146a/b expression seems to be increased compared to T0. Is that statistically significant? If yes, any explanation?

The change in miR-146a/b expression after 24 h removal of IL-1β is not statistically significant. We have added statistical analyses of the data presented in Figure 2 to aid in the interpretation of these findings.

4. Fig 3A. Over-expression of miR146a increased NOS3 mRNA expression, which contrasts with the down-regulation of adhesion molecule genes. Even though the explanation might not be easy, the authors should discuss this finding in the Discussion section.

We have added a substantial amount of new data that addresses the mechanisms of eNOS regulation by miR-146. We found that miR-146 regulated HuR expression (Fig. 7C), and that knock-down of HuR resulted in elevation of eNOS mRNA/protein (Fig. 7F,G). Therefore, in cells treated with miR-146a mimic, we anticipate that eNOS levels are elevated because of the diminished levels of HuR protein. We present data that demonstrates that HuR does not directly bind to eNOS mRNA (Fig. S8A). We believe that HuR regulates eNOS transcription by controlling the expression of KLF2, as shown previously by Rhee *et al, Proc Nat Acad Sci*, 2010. Indeed we found that *KLF2* was bound by HuR *in vitro* (Fig. S12A), and that *KLF2* levels increased in *HuR* knock-down cells (Fig. S12B).

5. Fig 8A. miR146a/b expression was analysed in the heart. Is miR146a/b expressed by cardiomyocytes? Expression in the aorta should be reported. What about the expression in low- and high-flow region (curvature vs descending aorta)?

We have included data on the expression of miR-146a/b in endothelial cells lining the descending aorta and find that expression is enriched in the endothelium compared to the cells in the wall of the vessel (Fig. 8A). This demonstrates that miR-146a/b is enriched in the intima, where it can regulate endothelial activation, but miR-146a/b is also expressed in the vessel wall. We have not assessed the expression of miR-146a/b in the low- and high-flow regions of the aorta, but we will be pursuing this line of investigation, since this will be important in studies of chronic inflammation. We do not believe that this data is relevant for the current manuscript, which focuses on models of acute inflammation. We also have not directly measured the expression of miR-146a/b in cardiomyocytes, although others have shown that miR-146a can be expressed in cardiomyocytes (Horie *et al, Cardiovasc Res*, 2010). However, we include immunofluorescence images of Vcam-1 expression in wild-type and *miR-146a*$^{-/-}$ mice treated with IL-1β, which shows that Vcam-1 is induced in the endothelium as well as in regions directly adjacent to the endothelium in knock-out mice treated with IL-1β (Fig. 8F). We did not observe Vcam-1 induction in cardiomyoctyes (data not shown). This suggests that even if miR-146a/b is expressed in cardiomyocytes, it does not play a role in the regulation of leukocyte adhesion molecules such as Vcam-1, E-Selectin and Icam-1, which are almost exclusively expressed in the endothelium.

6. Fig 8 C. HuR mRNA expression is reported. What about HuR protein expression?

We have now included HuR protein expression measurements in wild-type and *miR-146a*$^{-/-}$ mice (Fig. 8C), which suggests that HuR is a target of miR-146a *in vivo*.

7. Was NOS3 expression altered in miR146a-/- mice ? How was its expression modulated by ILb injection in WT and miR146a-/- mice? Same for EGR3.

We have included analyses of eNOS expression at the mRNA and protein level in wild-type and *miR-146a*$^{-/-}$ mice (Fig. S11; see response to comment #2). We have also included data on *Egr-1* and *Egr-3* expression (Fig. 8D), which demonstrates that these transcription factors are significantly elevated after a 2 h IL-1β treatment of *miR-146a*$^{-/-}$ mice.

Minor comment.

The Introduction is much too long.

We have decreased the length of the Introduction by approximately 350 words, as suggested by the Reviewer.

References:

Horie T, Ono K, Nishi H, Nagao K, Kinoshita M, Watanabe S, Kuwabara Y, Nakashima Y, Takanabe-Mori R, Nishi E, Hasegawa K, Kita T, Kimura T (2010) Acute doxorubicin cardiotoxicity is associated with miR-146a-induced inhibition of the neuregulin-ErbB pathway. Cardiovasc Res 87: 656-664

Knowles JW, Reddick RL, Jennette JC, Shesely EG, Smithies O, Maeda N (2000) Enhanced atherosclerosis and kidney dysfunction in eNOS(-/-)Apoe(-/-) mice are ameliorated by enalapril treatment. J Clin Invest 105: 451-458

Oemar BS, Tschudi MR, Godoy N, Brovkovich V, Malinski T, Lüscher TF (1998) Reduced endothelial nitric oxide synthase expression and production in human atherosclerosis. Circulation 97: 2494-2498

Rhee WJ, Ni CW, Zheng Z, Chang K, Jo H, Bao G (2010) HuR regulates the expression of stress-sensitive genes and mediates inflammatory response in human umbilical vein endothelial cells. Proc Natl Acad Sci USA 107: 6858-6863.

Weyrich AS, Buerke M, Albertine KH, Lefer AM (1995) Time course of coronary vascular endothelial adhesion molecule expression during reperfusion of the ischemic feline myocardium. J Leukoc Bio 57: 45-55

2nd Editorial Decision 22 April 2013

Thank you for the submission of your revised manuscript to EMBO Molecular Medicine. We have now received the enclosed reports from the Reviewers that were asked to re-assess it. As you will see the reviewers are now supportive and I am pleased to inform you that we will be able to accept your manuscript pending the following final amendments:

1) As per our Author Guidelines, the description of all reported data that includes statistical testing must state the name of the statistical test used to generate error bars and P values, the number (n) of independent experiments underlying each data point (not replicate measures of one sample), and the actual P value for each test (not merely 'significant' or 'P < 0.05').

2) Would you consider shortening your title slightly to increase impact? I would suggest something like: "MicroRNA-146 represses endothelial activation by inhibiting pro-inflammatory pathways" but please feel free to suggest an alternative.

Please submit your revised manuscript within two weeks. Needless to say, the sooner we receive it the sooner I will be able to formally accept your manuscript.

I look forward to reading a new revised version of your manuscript as soon as possible.

***** Reviewer's comments *****

Referee #1 (Comments on Novelty/Model System):

The authors have now provided compelling evidence for a unique role for miR-146a in the vascular endothelium (as opposed to leukocytes) by demonstrating differential roles for regulating TRAF6 vs HuR. Specifically, HuR deficiency interestingly led to induction of eNOS, but had no effect on EC adhesion molecules in response to cytokine stimulation. This is a critical point and provides insights defining a role for this factor in ECs. They have also adequately responded to the rest of the concerns raised.

Referee #1 (General Remarks):

The authors have nicely addressed all concerns raised.

Referee #3 (Comments on Novelty/Model System):

The authors correctly address my previous comments.
Novel data on post-transcriptional regulation of endothelial inflammation

Referee #3 (General Remarks):

No comments

2nd Revision - authors' response 26 April 2013

Response to Editorial Comments:

1) As per our Author Guidelines, the description of all reported data that includes statistical testing must state the name of the statistical test used to generate error bars and P values, the number (n) of independent experiments underlying each data point (not replicate measures of one sample), and the actual P value for each test (not merely 'significant' or 'P < 0.05').

We have now revised the Figures and Figure Legends to include information regarding the statistical test used, the number of independent experiments performed and the actual p-value. We have also included more information regarding the statistical tests that we utilized in the Methods section.

2) Would you consider shortening your title slightly to increase impact? I would suggest something like: "MicroRNA-146 represses endothelial activation by inhibiting pro-inflammatory pathways" but please feel free to suggest an alternative.

The change in title that you have suggested is agreeable to us.
