# Supplementary material for: MicroRNA-146 represses endothelial activation by inhibiting pro-inflammatory pathways
Source: EMBO Mol Med. 2013 Jun 3;5(7):949–66. doi: 10.1002/emmm.201202318 (PMC3721471; doi:10.1002/emmm.201202318)
Supplement: Supplementary file 2 [file emmm0005-0949-SD2.pdf]

Supporting Information:

Cheng *et al.,* MicroRNA-146 represses endothelial activation by inhibiting pro-inflammatory pathways

Table of Contents:

Supplemental Methods

Supporting Information Table I: Primers used for qRT- PCR

Supporting Information Figure S1: TNF-α induces miR-146a and miR-146b expression

Supporting Information Figure S2: Over-expression of miR-146a inhibits monocyte adhesion to IL-1β- treated bovine aortic endothelial cells (BAEC)

Supporting Information Figure S3: miR-146a does not directly regulate *EGR-3*

Supporting Information Figure S4: A potential miR-146 binding site in *HuR* is highly conserved across species

Supporting Information Figure S5: miR-146 controls the expression of *HuR* mRNA

Supporting Information Figure S6: *HuR* knock-down represses THP-1 adhesion to TNF-α-treated endothelial cells

Supporting Information Figure S7: Pedicted AU-rich elements (AREs) in the 3' UTRs of genes involved in endothelial activation

Supporting Information Figure S8: HuR binds to *VCAM-1* and *MCP-1* mRNA but does not regulate the induction of these genes by IL-1β

Supporting Information Figure S9: The miR-146 targets, *HuR* and *TRAF6*, have divergent effects on the induction of inflammatory genes

Supporting Information Figure S10: MicroRNAs previously implicated in regulating inflammation are not appreciably altered in *miR-146a*-/- mice

Supporting Information Figure S11: Expression of eNOS is modestly decreased in *miR-146a*-/- mice

Supporting Information Figure S12: *KLF2* mRNA is bound by HuR and knock-down of *HuR* leads to increased levels of *KLF2* transcripts

Supporting Information Figure S13: Schematic of a miR-146 feedback loop that controls endothelial activation

Supplemental Methods:

Luciferase assays and cloning: Constructs containing the wild-type *TRAF6* 3' UTR or a *TRAF6* 3' UTR with one of the two miR-146 binding sites mutated (in pMIR-REPORT) were previously described (Taganov et al, 2006). A 600-bp region of the 3' UTR of human *EGR3*, which contains the potential miR-146 binding site, was PCR amplified from HUVEC cDNA using the following primers: 5'-TAGAAGGAGAGAGAAGAAGATGAAGTTTGC and 5'-GAATTTCACC TTTTCACAATATCAAGCATA (with XbaI linkers), and was cloned into the XbaI site located in the 3' UTR of pGL3-promoter (Promega). Similarly a 517-bp region of the 3' UTR of human *HuR* (*ELAVL1)* was amplified using the following primers: 5'-GAGGCGTAAAATGGCTCTGT and 5'-AGTTACAGGCTGGTGGCTTT (with XbaI linkers). The miR-146 seed match in the *HuR* 3' UTR (AGTTCTC) was mutated to (ACAAGAC) by site-directed mutagenesis (QuikChange II Kit, Agilent). To generate a luciferase construct that included a concatemer of the potential miR-146 binding site in the 3' UTR of *EGR3*, the following oligos (containing a 5' phosphate group) were synthesized: 5'-GGGAGTTTTCCTTTG TTTTAATAAAACTGTT CTCAGACATTA, 5'-CCTAATGTCTGAGAACAGTTTTATTAA AACAAAGGAAAACTC; miR-146 seed match underlined). These oligos were annealed together, ligated using T4 ligase (since they contain CC and GG over-hangs, respectively), and run on an agarose gel. The band corresponding to 5 copies of the sequence was gel purified, blunt-end filled using DNA polymerase and blunt-end cloned into the XbaI site of pGL3. Oligonucleotides containing a mutated miR-146 binding site were also cloned (5'-GGGAGTTTTCCTTTGTTTTAATAAA ACTGTAGACAGACATTA and 5'-CCTAATGTCTGTCTACAGTTTTATTAAAACAAAGGA AAACTC; mutated miR-146 seed match underlined). The sequence, directionality and the number of concatemers inserted were confirmed by DNA sequencing.

HeLa cells grown in 12-well dishes were transfected with 1 μg of luciferase construct, 100 ng of pRL *Renilla* luciferase construct (Promega) (for normalization of transfection efficiency), and 20 nM of control or miR-146a mimic (Dharmacon), using Lipofectamine 2000. Cellular lysates were isolated 24 h post-transfection using Passive Lysis Buffer and luciferase activity was monitored using the Dual Luciferase Reporter Assay System (Promega) using a GloMax 20/20 Luminometer (Promega).

A *miR-146b* promoter/reporter construct (containing a 1 kb fragment of the *miR-146b* proximal promoter) was a kind gift from Dr. E. Flemington (Tulane University Health Sciences Center). Site-directed mutagenesis was used to delete a putative EGR binding site 858-848 bp upstream of the mature miR-146b sequence using the following primers: 5'-GGGTTCCTG GCCCCCTTCCTCCTTTC and 5'-GAAAGGAGGAAGGGGGCCAGGAACCC. HeLa cells were transfected with 1 μg of wild-type or EGR-deleted *miR-146b* promoter/luciferase constructs together with a 0.5 μg of an empty or EGR3 expression construct (a kind gift from Dr. J.D. Powell (John Hopkins)) as above. A *Renilla* construct (100 ng) was co-transfected to control for transfection efficiency. To analyze NF-κB activity, HUVEC were first transfected with control, miR-146a mimic, miR-146 inhibitor, *TRAF6* or *HuR* siRNAs, and after 24 h the cells were electroporated with 1 μg of a 5x NF-κB element-luciferase reporter (Promega) and 0.5 μg of Renilla (to control for electroporation efficiency) using a Lonza 4D Nucleofector with the P5 Primary Cell Kit. After 24 h, cells were treated with 10 ng/mL of IL-1β for 6 h, and luciferase activity was assessed as above.

Supporting Information Table I: Primers used for qRT- PCR

**Gene**	**Forward Primer (5' -> 3')**	**Reverse Primer (5' -> 3')**
human *VCAM1*	GTTGAAGGATGCGGGAGTAT	GGATGCAAAATAGAGCACGA
human *SELE* (E-Selectin)	CTGGCCTGCTACCTACCTGT	AGCTACCAAGGGAATGTTGG
human *ICAM1*	CGGCCAGCTTATACACAAGA	GTCTGCTGGGAATTTTCTGG
human *CCL2* (MCP-1)	TCATAGCAGCCACCTTCATT	CGAGCCTCTGCACTGAGAT
human *EGR1*	CAGCACCTTCAACCCTCAG	TAACTGGTCTCCACCAGCAC
human *EGR3*	ACAATCTGTACCCCGAGGAG	GTAAGAGAGTTCCGGGTTGG
human *pri-miR-146a* (exon 1/intron 1)	CGGCTGAATTGGAAATGATA	TGCTGCCTCTCAAACAGAAG
human *pri-miR-146b*	AAGAAAGCATGCAAGAGCAG	GCCTTGGCATTGATGTTGTA
human *c-FOS*	TACTACCACTCACCCGCAGA	AGTGACCGTGGGAATGAAGT
human *c-JUN*	GAGAGCGGACCTTATGGCTA	GTGAGGAGGTCCGAGTTCTT
human *NOS3* (eNOS)	GGCATCACCAGGAAGAAGACC	TCACTCGCTTCGCGATCAC
human *TRAF6*	CCAAATCCATGCACATTCA	TTCTCATGTGTGACTGGGTGT
human *ELAVL1* (HuR)	CTCTCGCAGCTGTACCACTC	CACGTTGACGCCAGAGAG
human KLF2	Taqman assay #Hs00360439_g1	
human *GAPDH*	AGGTGAAGGTCGGAGTCAAC	GAGGTCAATGAAGGGGTCAT
human *TBP*	TCG GAG AGT TCT GGG ATT GT	CAC GAA GTG CAA TGG TCT TT
mouse *Vcam1*	GCACAAAGAAGGCTTTGAAGCA	GATTTGAGCAATCGTTTTGTATTCAG
mouse *Sele* (E-Selectin)	GAACCAAAGACTCGGGCATGT	ATGACCACTGCAGGATGCATT
mouse *Icam1*	CTGCCTTGGTAGAGGTGACTGA	AGGACAGGAGCTGAAAAGTTGTAGA
mouse *Ccl2* (Mcp-1)	GTCCCTGTCATGCTTCTGG	ATTGGGATCATCTTGCTGGT
mouse *Egr-1*	CTACCAATCCCAGCTCATCAAAC	CTCATCCGAGCGAGAAAAGC
mouse *Egr-3*	AAGCCCTTTGCCTGTGAGTTC	CGACTTCTTCTCCTTTTGCTTGA
mouse *Elavl1* (HuR)	GTACACCACCAGGCACAGAG	CCAAGGTTGTAGATGAAGATGC
mouse *NOS3* (eNOS)	CCAAGGTGATGAGCTCTGTG	GAAGATATCTCGGGCAGCAG
mouse *Tbp*	ACCCACCAGCAGTTCAGTAC	CTGCTCTAACTTTAGCACCTGT

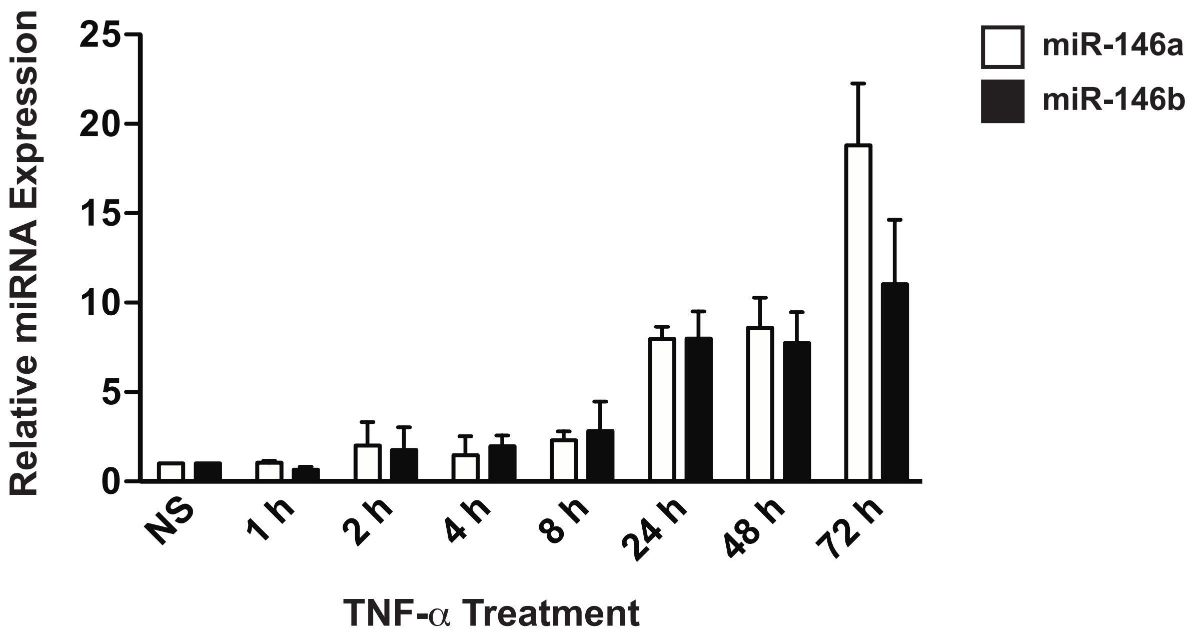

Supporting Information Figure 1: TNF-α induces miR-146a and miR-146b expression. HUVEC were stimulated with TNF-α and expression of miR-146a and miR-146b was assessed by qRT-PCR. Shown is the mean +/- SEM of 2 independent experiments. NS = non-stimulated.

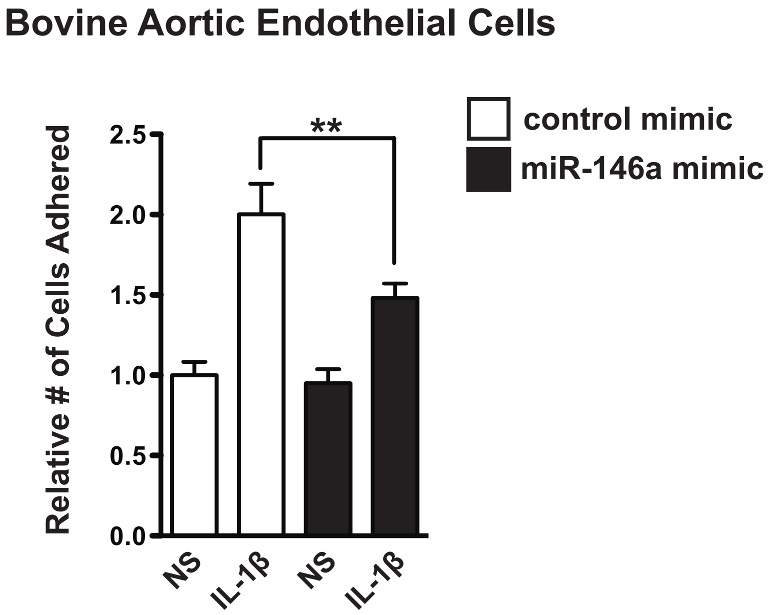

Supporting Information Figure 2: Over-expression of miR-146a inhibits monocyte adhesion to IL-1β-treated bovine aortic endothelial cells (BAEC). Shown is a representative experiment (quantification of 3 images from 3 indendent wells). ANOVA, $p<0.0001$. ** indicates a significant decrease ($p<0.01$) in the number of cells adhered to IL-1β-treated BAEC transfected with miR-146a mimic compared to control mimic.

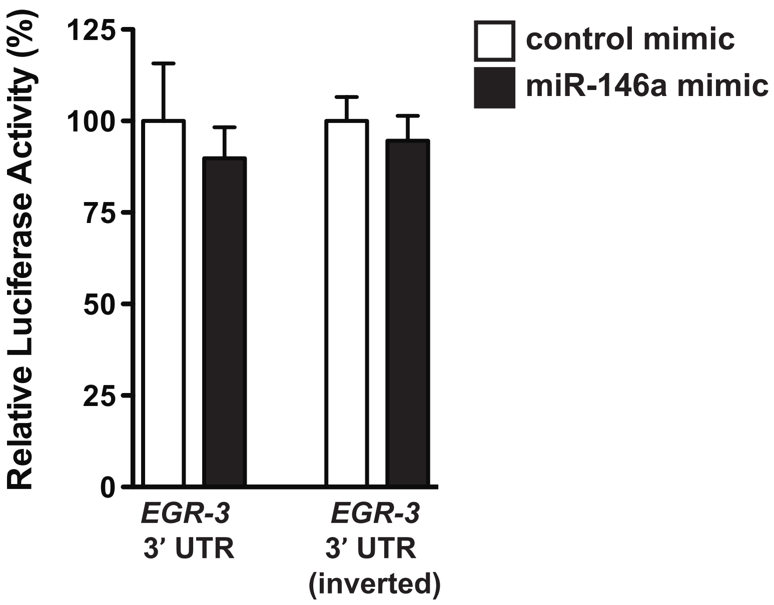

Supporting Information Figure 3: miR-146a does not directly regulate *EGR-3* - A Luciferase construct containing a fragment of the *EGR-3* 3' UTR that includes a putative miR-146 binding site was transfected into HeLa cells together with control or miR-146a mimic, and luciferase activity was measured. No change in luciferase activity was observed. As a control, a luciferase construct containing the same *EGR-3* 3' UTR, but in the inverse orientation, was used. Data from a representative experiment (transfections performed in triplicate) is shown.

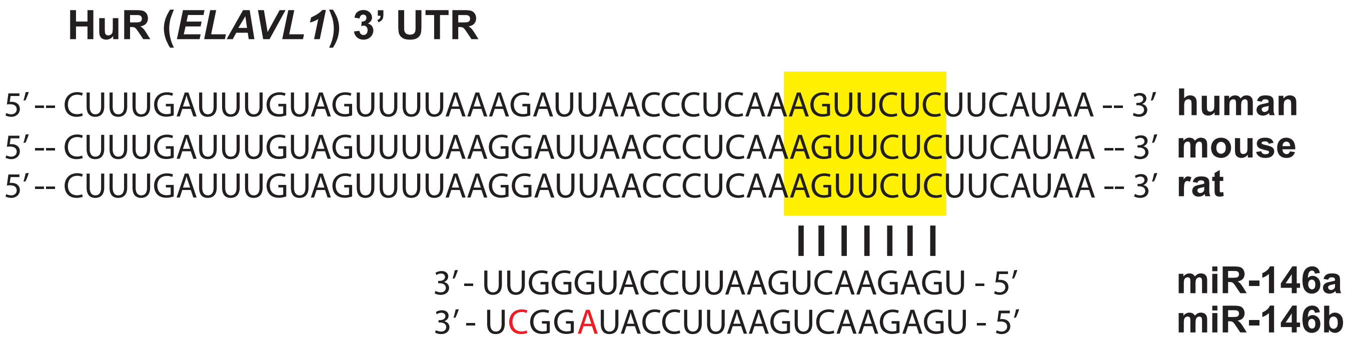

Supporting Information Figure 4: A potential miR-146 binding site in *HuR* is highly conserved across species - A portion of the 3' UTR of *HuR* is shown from human, mouse and rat, with the potential miR-146 binding site highlighted in yellow. The sequence of miR-146a and miR-146b is shown below, with the sequence differences between miR-146a and miR-146b indicated in red. The sequence of human, mouse and rat miR-146a and miR-146b are identical to that shown.

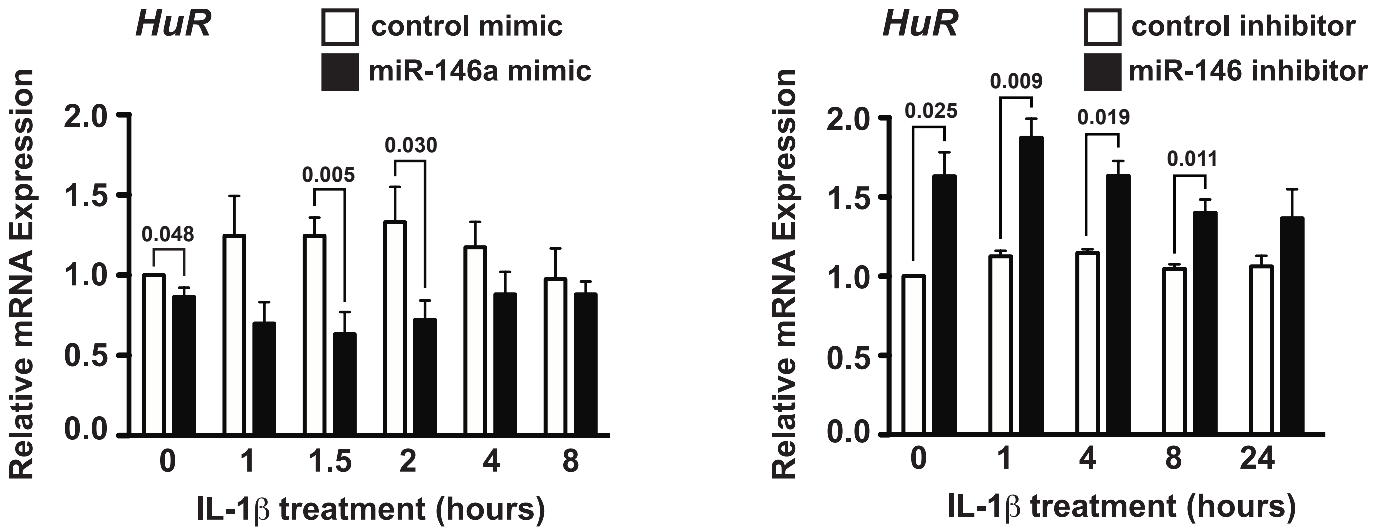

Supporting Information Figure 5: miR-146 controls the expression of *HuR* mRNA. Over-expression of miR-146a in endothelial cells reduced levels of *HuR* mRNA in IL-1β-treated HUVEC (left), as assessed by qRT-PCR, while inhibition of miR-146 increased *HuR* mRNA (right). p-values of significant differences (t-test) are indicated above (n = 4-5).

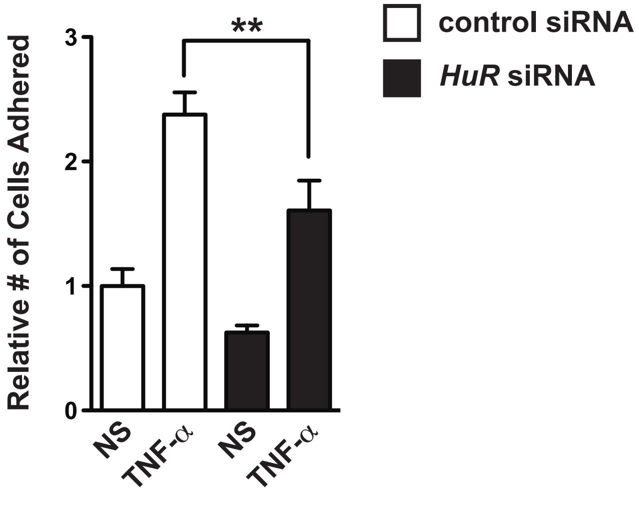

Supporting Information Figure 6: *HuR* knock-down represses THP-1 adhesion to TNF-α-treated endothelial cells - Shown is a representative experiment (quantification of 3 images in 3 independent wells). ANOVA, p<0.0001. ** indicates a significant decrease (p<0.01) in THP-1 adhesion to TNF-α-treated HUVEC transfected with *HuR* siRNA compared to control siRNA.

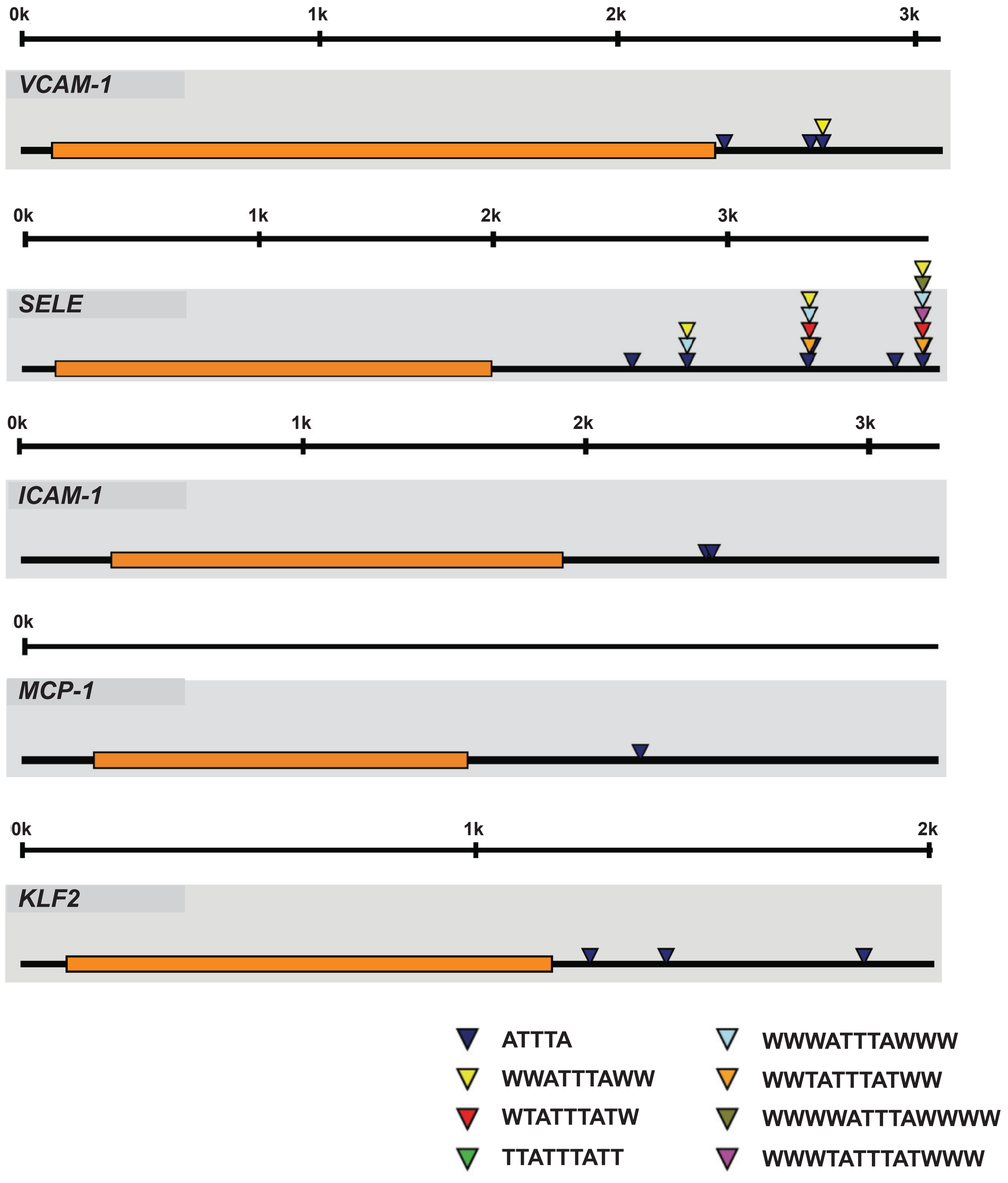

Supporting Information Figure 7: Pedicted AU-rich elements (AREs) in the 3' UTRs of genes involved in endothelial activation - Prediction of AREs was performed using AREsite (Gruber *et al*, *Nucleic Acids Research*, 2010). The coding region of each transcript is indicated in orange. The various types of AREs are indicated by colored trianges.

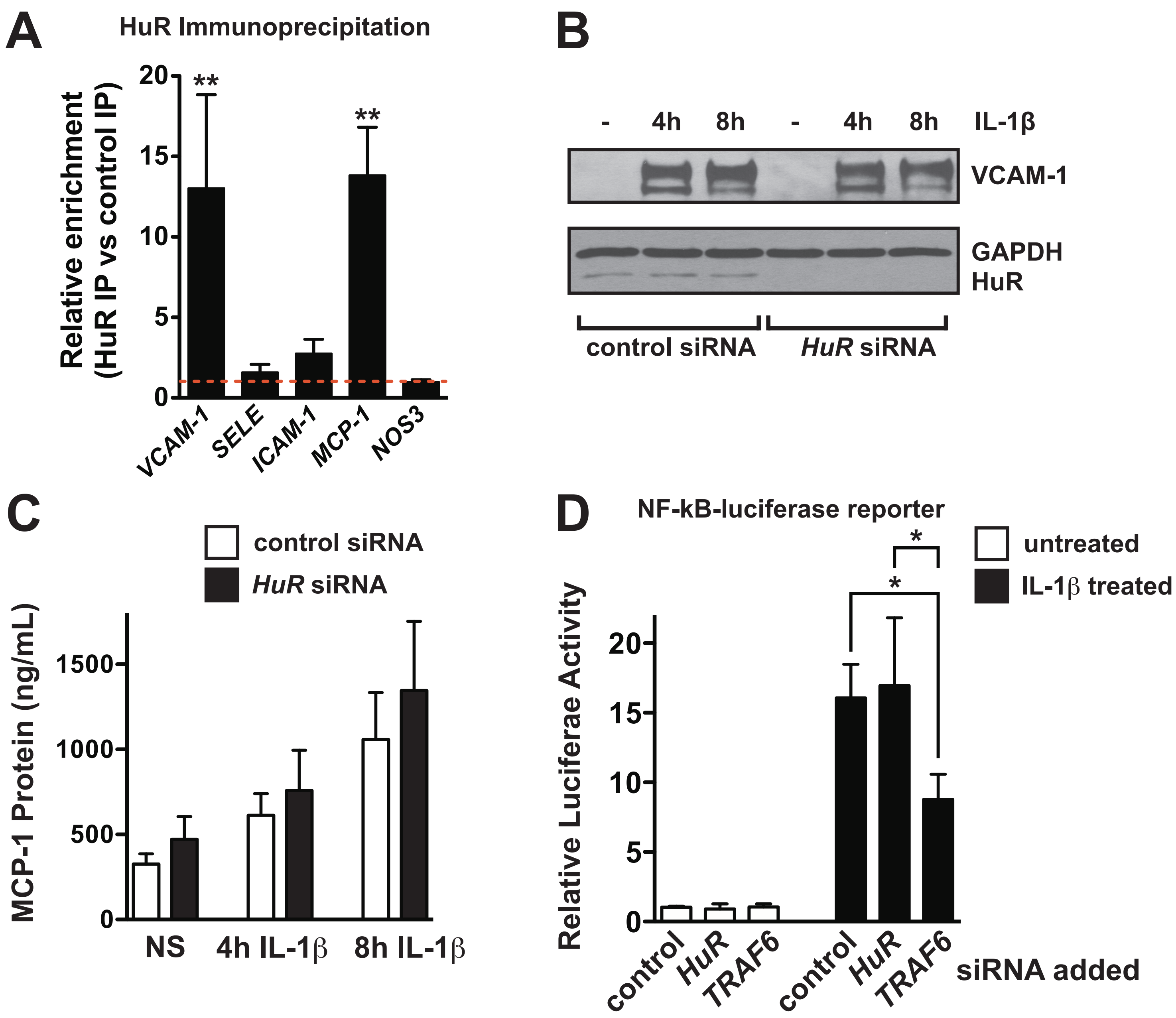

Supporting Information Figure 8: HuR binds to *VCAM-1* and *MCP-1* mRNA but does not regulate the induction of these genes by IL-1β - (A) HuR was immunoprecipitated from IL-1β-treated endothelial cells (4 h), RNA was isolated and the expression of several inflammatory genes and eNOS (*NOS3*) was assessed by qRT-PCR. Control immunoprecipitation was performed using an antibody to V5. *VCAM-1* and *MCP-1* were significantly enriched in HuR immunoprecipitates compared to V5 immunoprecipitates (n = 4). Repeated measures ANOVA, $p=0.0021$. ** indicates a significant difference compared to V5 immunoprecipitation, $p<0.01$. **(B)** Expression of VCAM-1 in response to IL-1β treatment was not affected by *HuR* knock-down. A representative blot is shown. **(C)** Expression of MCP-1 was not affected by HuR knock-down, as assessed by ELISA. Shown is the mean +/- SEM (n = 3). **(D)** Activation of NF-κB signaling was not affected by *HuR* knock-down, but was significantly decreased in *TRAF6* knock-down cells, as assessed by NF-κB-luciferase reporter assay (n = 4). Repeated measures ANOVA, $p<0.0001$. * indicates a signficant difference, $p<0.05$.

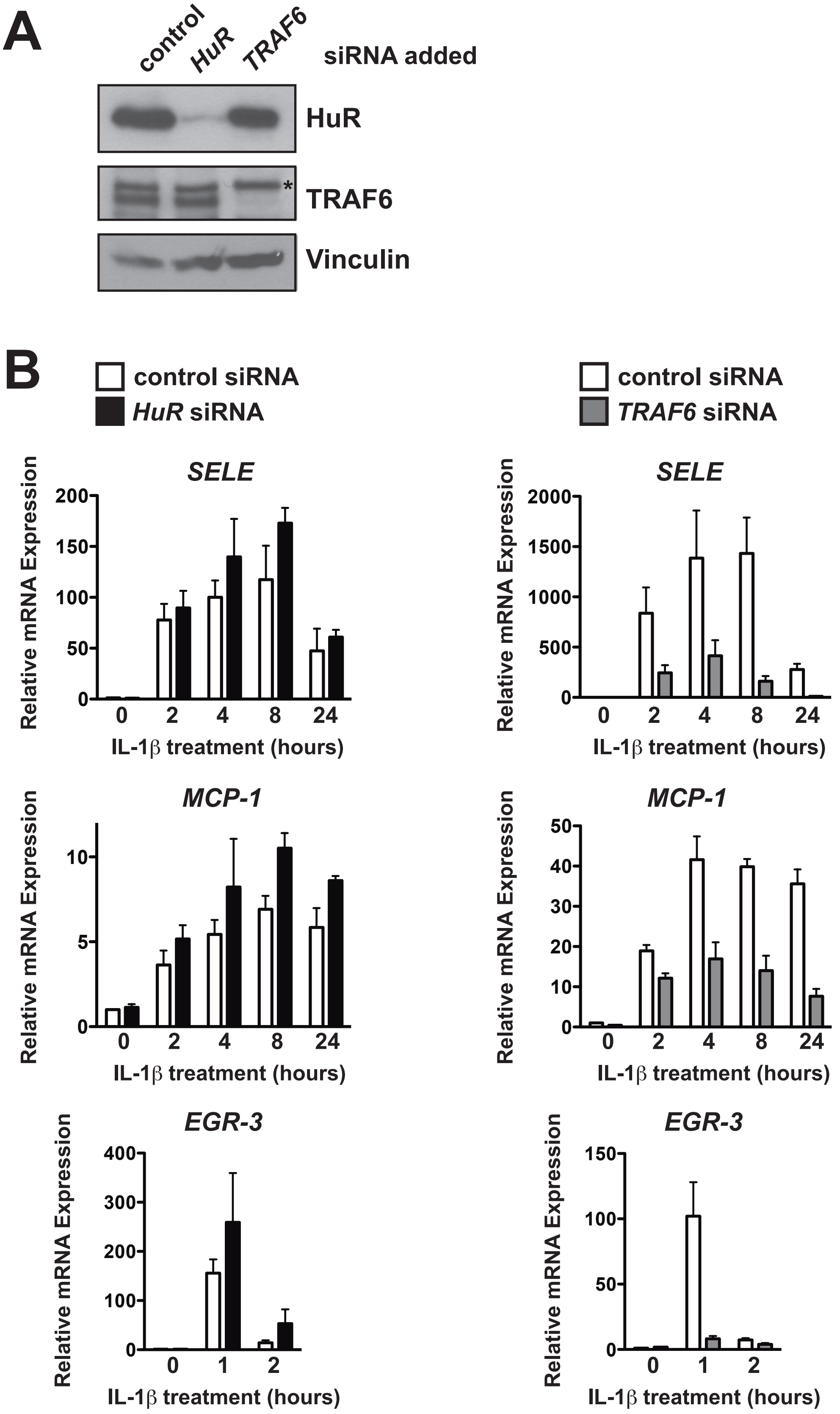

Supporting Information Figure 9: The miR-146 targets, *HuR* and *TRAF6*, have divergent effects on the induction of inflammatory genes - (A) Western blot demonstrating the efficient knock-down of HuR or TRAF6. Vinculin was used as a loading control. * indicates a non-specific band. **(B)** The induction of *SELE*, *MCP-1* and *EGR-3* was assessed in *HuR* (left) or *TRAF6* (right) knock-down cells in response to IL-1β treatment. While *TRAF6* knock-down decreased the induction of these genes, *HuR* knock-down had no effect (n = 3).

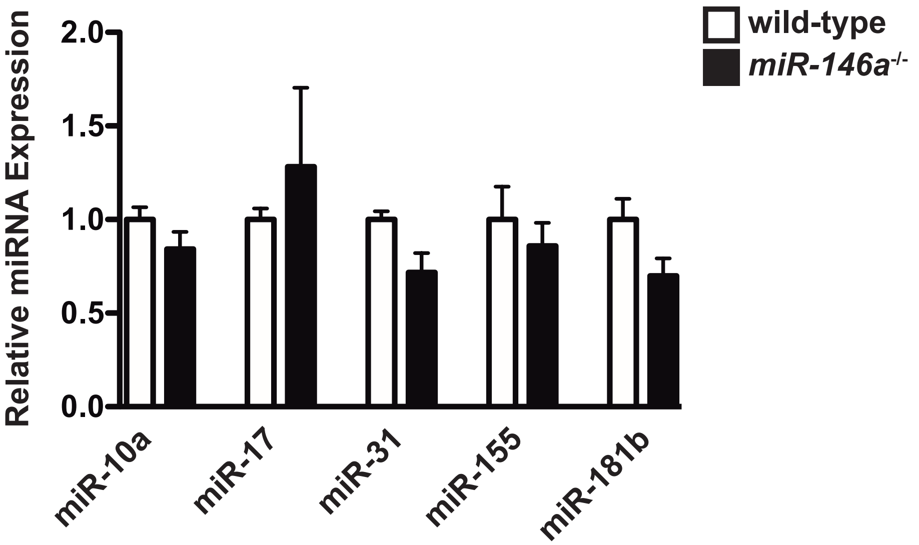

Supporting Information Figure 10: MicroRNAs previously implicated in regulating inflammation are not appreciably altered in *miR-146a*$^{-/-}$ mice - MicroRNA expression was assessed in the hearts of wild-type and *miR-146a*$^{-/-}$ mice (3-4 months of age) by qRT-PCR. Data was normalized to the expression of miR-126 (n = 6).

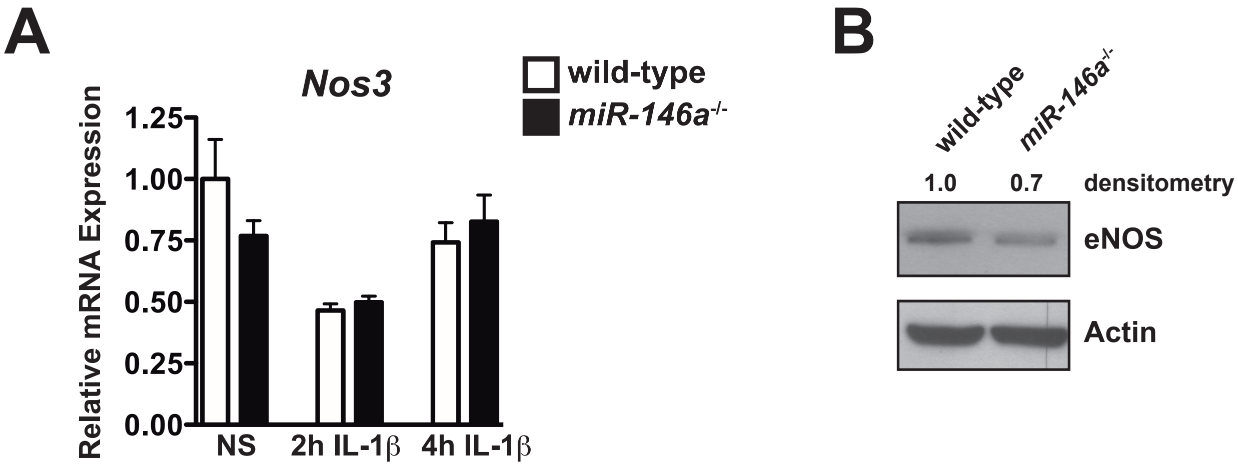

Supporting Information Figure 11: Expression of eNOS is modestly decreased in *miR-146a*$^{-/-}$ mice - (A) *Nos3* (eNOS) mRNA expression was assessed by qRT-PCR in wild-type and *miR-146a*$^{-/-}$ hearts, revealing a trend towards decreased levels in knock-out mice (n = 3-6). NS = non-stimulated. **(B)** Expression of eNOS protein was assessed in wild-type and *miR-146a*$^{-/-}$ hearts by western blot. A representative blot is shown.

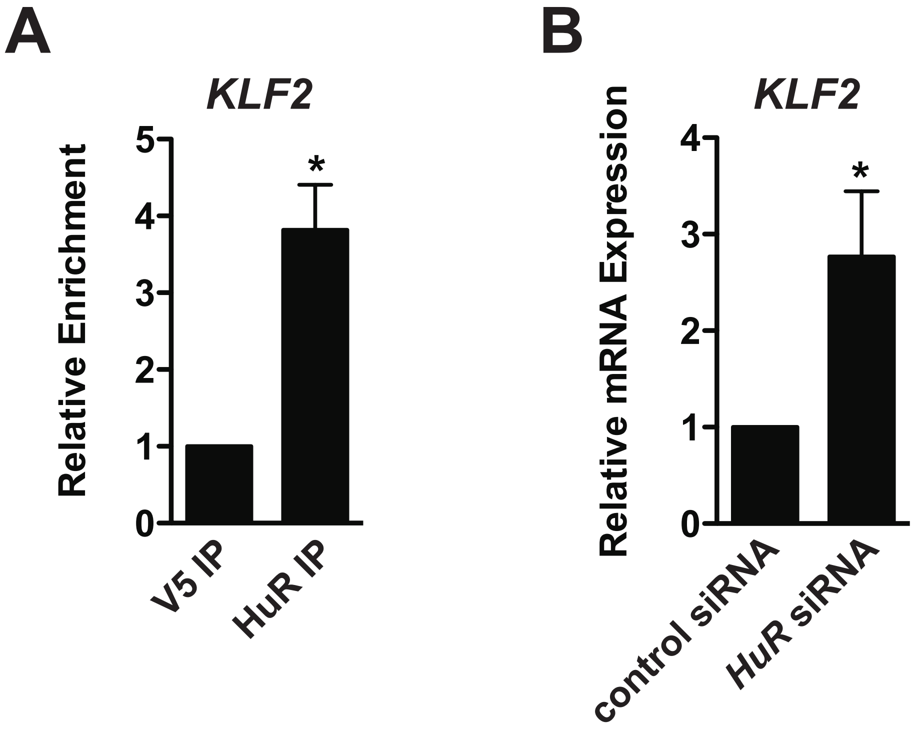

Supporting Information Figure 12: *KLF2* mRNA is bound by HuR and knock-down of *HuR* leads to increased levels of *KLF2* transcripts - (A) *KLF2* mRNA was enriched in HuR immunoprecipitates from unstimulated HUVEC compared to control immunoprecipitates (V5), as assessed by qRT-PCR (t-test, p=0.017, n = 4). **(B)** *KLF2* mRNA was increased in HUVEC transfected with *HuR* siRNA (t-test, p=0.030, n = 5).

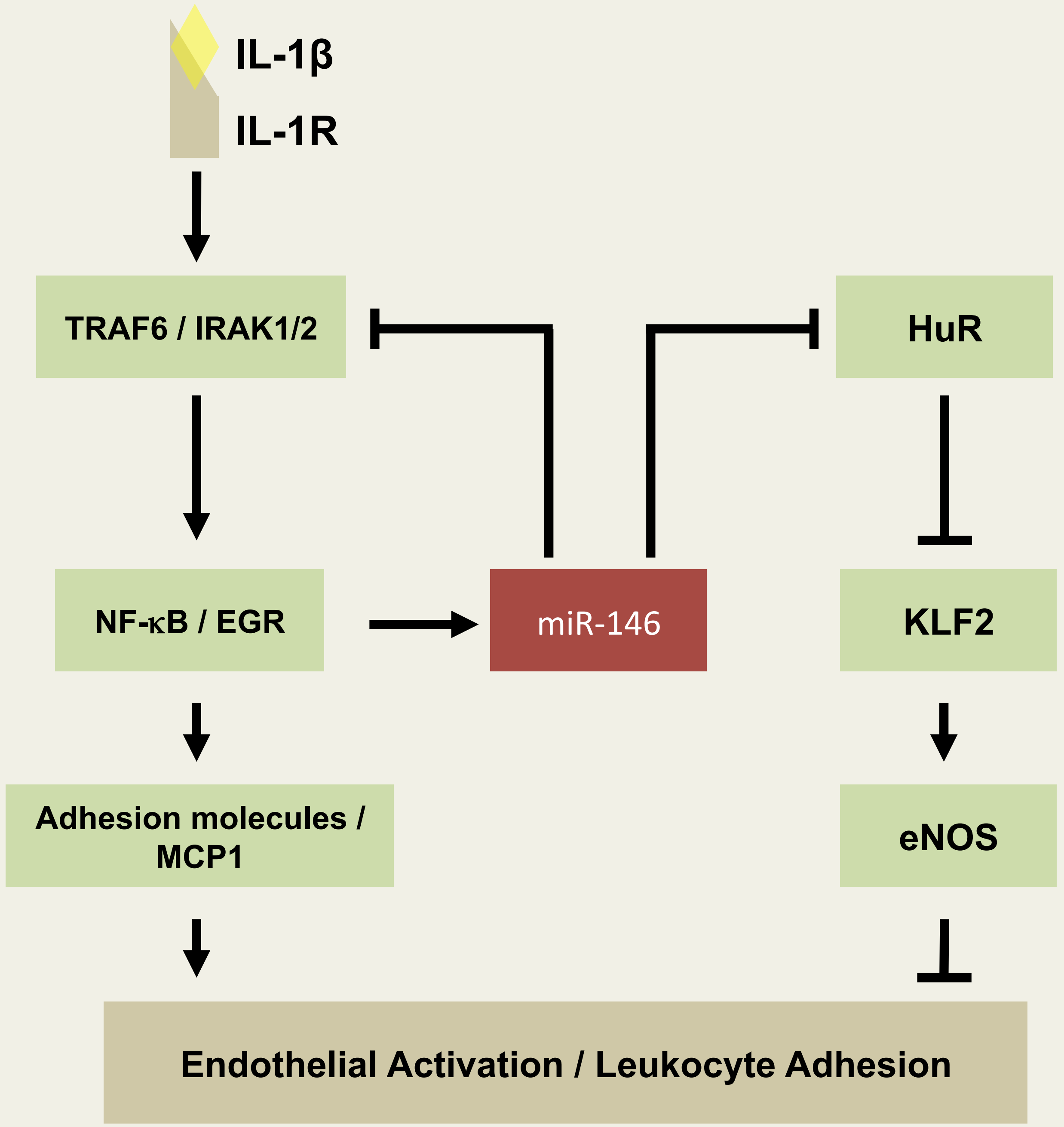

Supporting Information Figure 13: Schematic of a miR-146 feedback loop that controls endothelial activation. Pro-inflammatory cytokines activate the NF-κB and EGR transcription factors, which induce the expression of leukocyte adhesion molecules and chemokines, such as MCP-1. These pathways also induce the expression of miR-146, which targets TRAF6 and IRAK1/2, and functions as a negative regulator that represses inflammatory signaling. MiR-146 also targets HuR, which represses KLF2, a potent transcriptional activator of eNOS. Nitric oxide produced by eNOS is a vasodilator and a repressor of leukocyte and platelet adhesion to the endothelium.
